# The laboratory investigation, management, and infection prevention and control of Candida auris: a narrative review to inform the 2024 national guidance update in England

**DOI:** 10.1099/jmm.0.001820

**Published:** 2024-05-21

**Authors:** Christopher R. Jones, Claire Neill, Andrew M. Borman, Emma L. Budd, Martina Cummins, Carole Fry, Rebecca L. Guy, Katie Jeffery, Elizabeth M. Johnson, Rohini Manuel, Mariyam Mirfenderesky, Ginny Moore, Bharat Patel, Silke Schelenz, Karren Staniforth, Surabhi K. Taori, Colin S. Brown

**Affiliations:** 1 HCAI, Fungal, AMR, AMU, and Sepsis Division, UK Health Security Agency, London, UK; 2 UKHSA Mycology Reference Laboratory, National Infection Services, UKHSA South West Laboratory, Science Quarter, Southmead Hospital, Bristol, UK; 3 MRC Centre for Medical Mycology, University of Exeter, Geoffrey Pope Building, Stocker Road, Exeter, UK; 4 Department of Microbiology and Infection Control, Barts Health NHS Trust, London, UK; 5 Oxford University Hospitals NHS Foundation Trust, Oxford, UK; 6 Radcliffe Department of Medicine, University of Oxford, Oxford, UK; 7 Public Health Laboratory London, Science Group, UK Health Security Agency, London, UK; 8 Research and Evaluation, UK Health Security Agency, Porton Down, Salisbury, UK; 9 Department of Microbiology, King’s College Hospital NHS Foundation Trust, London, UK; 10 Department of Medical Microbiology, NHS Lothian, Edinburgh, UK; 11 National Institute for Health Research Health Protection Research Unit (NIHR HPRU) in Healthcare Associated Infections and Antimicrobial Resistance, Imperial College London, London, UK

## Abstract

The emergent fungal pathogen *Candida auris* is increasingly recognised as an important cause of healthcare-associated infections globally. It is highly transmissible, adaptable, and persistent, resulting in an organism with significant outbreak potential that risks devastating consequences. Progress in the ability to identify *C. auris* in clinical specimens is encouraging, but laboratory diagnostic capacity and surveillance systems are lacking in many countries. Intrinsic resistance to commonly used antifungals, combined with the ability to rapidly acquire resistance to therapy, substantially restricts treatment options and novel agents are desperately needed. Despite this, outbreaks can be interrupted, and mortality avoided or minimised, through the application of rigorous infection prevention and control measures with an increasing evidence base. This review provides an update on epidemiology, the impact of the COVID-19 pandemic, risk factors, identification and typing, resistance profiles, treatment, detection of colonisation, and infection prevention and control measures for *C. auris*. This review has informed a planned 2024 update to the United Kingdom Health Security Agency (UKHSA) guidance on the laboratory investigation, management, and infection prevention and control of *Candida auris*. A multidisciplinary response is needed to control *C. auris* transmission in a healthcare setting and should emphasise outbreak preparedness and response, rapid contact tracing and isolation or cohorting of patients and staff, strict hand hygiene and other infection prevention and control measures, dedicated or single-use equipment, appropriate disinfection, and effective communication concerning patient transfers and discharge.

## Data Summary

Data sharing is not applicable to this article as no datasets were generated or analysed during the current study.

## Introduction

*Candida auris* is an emerging fungal pathogen, first described in 2009 from the external auditory canal of a patient in Japan [1], but identified in retrospect as far back as 1996 [2]. It has now been detected worldwide, across 6 continents, and in over 60 countries [3]. The inaugural World Health Organization (WHO) fungal priority pathogen list considers *C. auris* a critical priority pathogen, highlighting its threat to global public health [4].

*C. auris* infections have been frequently reported from the bloodstream and seen in association with bone, central nervous system (CNS), and intra-abdominal infections [5]. It has additionally been isolated from wounds, ear and respiratory specimens, urine, bile, and jejunal biopsies [6]. Detection in surveillance swabs from the axilla and/or groin may indicate carriage rather than infection, with carriage posing a risk of transmission to others and of subsequent invasive infection. Worryingly, *C. auris* is highly transmissible between patients in healthcare settings and is associated with prolonged persistence in the environment [7,8]. Of further concern, accurate identification requires specialised laboratory methods, which are not available in many countries, thus challenging outbreak detection and control efforts.

There have been six genetically distinct clades of *C. auris* discovered to date. This includes the South Asian clade, which was first detected in India and Pakistan (clade I), the East Asian clade first detected in Japan (clade II), the South African clade first detected in South Africa (clade III), the South American clade first detected in Venezuela (clade IV), and two further clades; one recently detected in Iran (clade V) [9] and a novel clade VI in Singapore [10]. However, depending on population movement, these clades can be detected elsewhere. Antifungal resistance to fluconazole is common, and patterns of resistance to other antifungal agents vary by clade and region. Invasive infections from multidrug-resistant (MDR) *C. auris* have been associated with high mortality, and concerns have been raised regarding transmission, persistent colonisation, and the need for effective disinfection. Moreover, active community spread of independent clusters of MDR *C. auris* has now been described in the USA [11,12].

This critical-priority fungal pathogen is increasingly detected globally, including across Europe [13], and is considered endemic in some countries such as the USA [14], South Africa [15], and India [16]. Given the limited mycology diagnostic capacity in many countries, it is likely that *C. auris* may be more widespread than previously thought [17]. This review provides an update on epidemiology, the impact of the COVID-19 pandemic, risk factors, identification and typing, resistance profiles, treatment, colonisation, and infection prevention and control measures. This review informed the recent update to the United Kingdom Health Security Agency (UKHSA) guidance on the laboratory investigation, management, and infection prevention and control of *Candida auris*.

## Methods

To identify updates since a previous review of the *C. auris* literature [18], a search of articles was conducted, between 2019 and 2023, with snowballing for any other significant studies. This included Medline, Embase, Scopus, NICE Evidence Search, Global Health, and CINAHL, and was limited to publications in the English language. The search terms *Candida auris* and *C. auris* were used. Articles were deduplicated and excluded if there was no reference to *C. auris* or if they did not contain information relevant to the key areas of the review, as described above.

### Emergence and spread of *C. auris*

*C. auris* was first detected in 2009 in Japan; however, the first retrospectively identified isolate dates back to 1996 [1,19]. In the Republic of Korea, 15 patients with samples taken between 2006 and 2011 were found to have *C. auris*, all with a background of chronic otitis media and systemic antifungal use [20]. Following additional epidemiological investigation, it was proposed that intra- and interhospital clonal transmission had occurred [2]. In Europe the first imported case dates to 2007, a South Indian clade (clade I) isolate [21].

Despite these discoveries, retrospective analyses of international samples support that *C. auris* was a novel pathogen prior to 2009. From 15 271 retrospectively analysed *Candida* isolates taken between 2004 and 2015, only 4 were found to be *C. auris*, all of which had been collected since 2009 [22]. A further retrospective analysis of unusual *Candida* species performed at the Mycology Reference Laboratory, UK Health Security Agency (UKHSA), failed to find any evidence of *C. auris* in the UK prior to 2013 [23].

The first reports of invasive fungaemia associated with *C. auris* were from a multicentre surveillance study of candidaemia conducted in the Republic of Korea in 2009, with one case dating back to 1996 from an incidental finding of unidentified yeasts in bloodstream isolates [19]. All cases had been hospitalised for at least 12 days before isolation of the organism. Invasive infections were also discovered in India dating back to 2009, initially in 12 inpatients from 2 hospitals in New Delhi, with clonal isolates suggesting interhospital transmission [24].

**Fig. 1.**
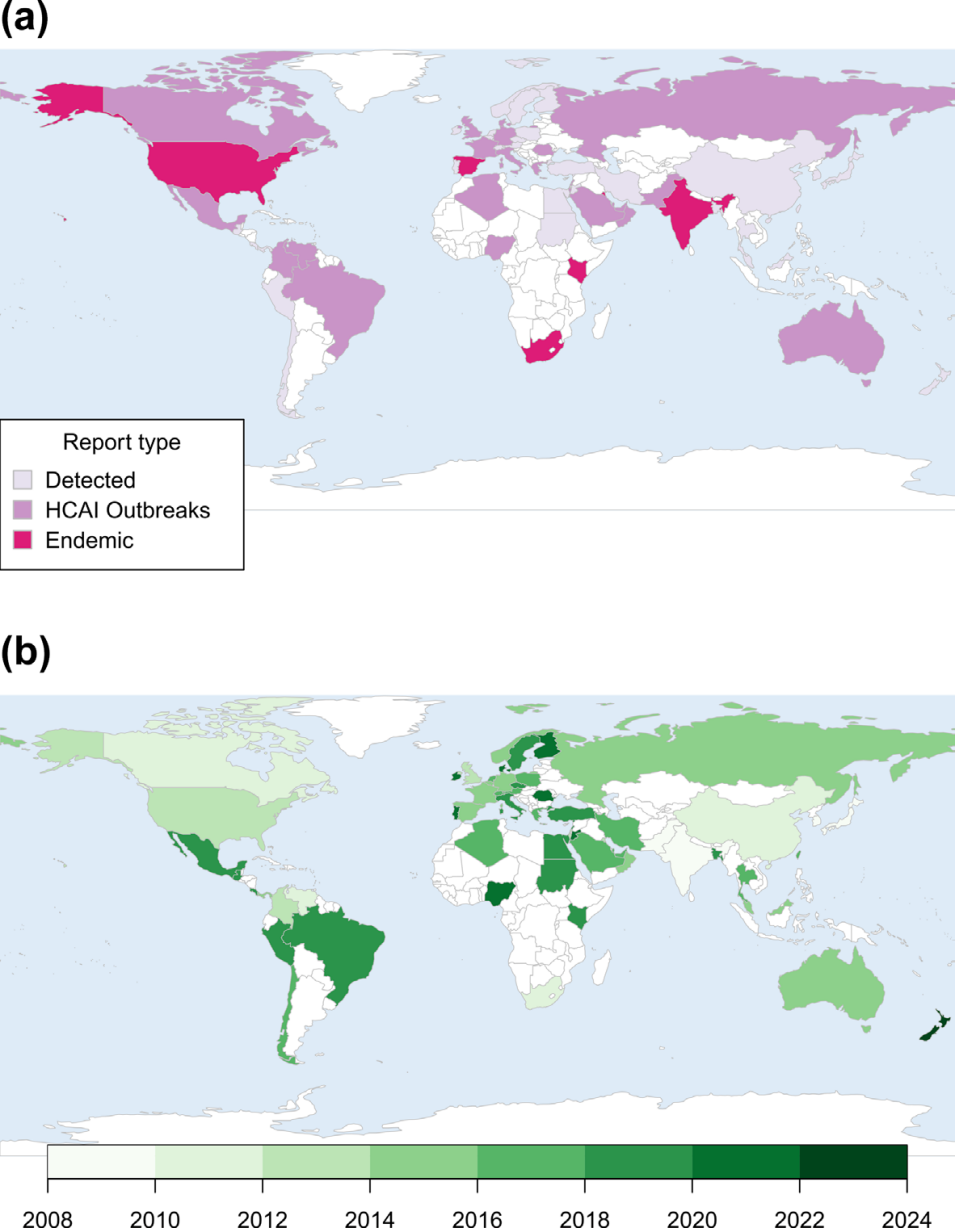
Global epidemiology of *Candida auris*. (**a**) Countries where *C. auris* has been reported are presented and categorised by reported association with HCAI outbreaks or evidence of endemicity within a country. (**b**) Countries where *C. auris* has been reported are presented according to the year that the first case was detected. Note: there are many countries where *C. auris* has not yet been reported (white shading); however, this does not mean that it is not present within these countries. Cases of *C. auris* have been detected and reported in Reunion; however, this is not visible on the maps. Maps were prepared in R (v4.3.1) using the package rworldmap. See Supplementary Material 1 for a full list of countries and references used to produce these maps.

As of December 2023, *C. auris* has been identified in 61 countries across 6 continents (Fig. 1, Supplementary Material 1). In the USA, *C. auris* became notifiable in 2018, and reports of case detection subsequently increased more than twofold. Between January and December 2022, 2377 clinical cases had been identified across the USA and targeted screening had identified a further 5754 screening cases [25]. In response to a substantial rise in detection in European countries, a rapid risk assessment of *C. auris* in healthcare settings was updated by the European Centre for Disease Prevention and Control (ECDC) in 2018 [26], with a further risk assessment in 2022 following an outbreak in northern Italy. Additionally, a survey by the ECDC in 2020 reported ongoing variability in laboratory capacity and overall preparedness for *C. auris* detection [27].

*C. auris* represents a significant burden of disease in certain countries and has become endemic in several [15,16]. In South Africa, the organism has been detected in almost 100 hospitals, causing large outbreaks, and is reportedly responsible for approximately 1 in 10 cases of candidaemia [15]. In India, *C. auris* has been implicated in 5 % of candidaemia cases across 27 intensive care units [28]. Healthcare outbreaks have been reported from hospitals in several countries, with even more reporting sporadic cases (Fig. 1, Supplementary Material 1). These reports likely represent under-ascertainment of *C. auris* globally, given the challenges in laboratory identification and lack of laboratory infrastructure in some countries.

Community prevalence of *C. auris* remains unknown, and screening on admission to hospital is not routine practice. Following three large outbreaks in acute healthcare settings, a surveillance study in England was performed in 2017–18, involving screening of 998 admissions to 8 intensive care units across 3 major cities; all screens were found to be negative [29]. Another study in the USA found that carriage was only detected in people previously exposed to the hospital environment [30]. In areas where *C. auris* is endemic, however, community cases may be more prevalent. A recent study looking at *C. auris* amongst patients with chronic respiratory disease in India found that 9.3 % (3/32) of patients who were colonised with *C. auris* were colonised at the time of admission [8].

### Genomic analysis and epidemiology

Whole-genome sequencing (WGS) analyses indicate that *C. auris* emerged simultaneously on several different continents, with four distinct clades across three continents; South Asian (clade I), East Asian (clade II), African (clade III), and South American (clade IV) [31]. A further genetically distinct clade has more recently been isolated in Iran from a 14-year-old girl who had never travelled abroad and was diagnosed with otomycosis, followed by a further fluconazole-resistant case distinct from the first [32,33]. A sixth clade has just been reported in patients from Singapore and Bangladesh [10].

Each of the clades are separated by thousands of single-nucleotide polymorphisms (SNPs), but strains are highly clonal within each clade, with on average fewer than 70 SNPs separating any 2 isolates [34]. This, along with various geographical resistance mechanisms, supports the hypothesis of independent clonal expansion and evolution. Potential drivers for the emergence of the pathogen include increasing antifungal selection pressures in humans, animals, and the environment [31].

A retrospective analysis of 912 worldwide cases across 44 countries between 2009 and 2020 found the South Asian strain (clade I) to be the most prevalent, having been identified in 17 countries [35]. This was followed by the South African strain (clade III) found in eight countries [35]. Only five countries reported presence of the East Asian strain (clade II) and three of the South American strain (clade IV).

### Experience in the UK

A retrospective analysis of historical isolates showed that the first identified *C. auris* isolates in the UK were in 2013, from blood cultures in unrelated patients [23]. In 2014, a single isolate from pleural fluid was recorded, and in 2015 there were 15 isolates identified, 9 of which were from sterile sites. Between 2013 and 2022, a total of 363 *C*. *auris* isolates were reported through laboratory surveillance in England, with 44 (12.1 %) isolated from blood culture specimens [36]. During the COVID-19 pandemic, the number of isolates reported reduced substantially, probably reflecting the lack of new introductions from endemic areas due to extreme travel restrictions. However, cases have now begun to increase, and new outbreaks have been detected in acute healthcare settings. All clades except the South American clade and recently discovered clades V and VI have been identified to date in the UK. Sporadic introductions continue, many involving patients repatriated directly from international hospitals in endemic regions, including India, Qatar, Kuwait, Oman, Pakistan, and Kenya [37].

Previously, outbreaks have been identified in three separate public sector hospitals in England, each of which was prolonged and challenging to control [38,41]. The first was in a cardiothoracic centre between 2015 and 2016, involving 50 patients over a 16 month period. Most cases were confined to colonisation of skin sites or mucosa, and 44 % (*n* = 22/50) required antifungal therapy [39]. The second outbreak started in 2016 and involved 34 patients including 8 bloodstream infections. It was controlled within 13 months by an intensive screening and infection control approach maintaining a low mortality rate [40]. The third outbreak from 2015 to 2017 involved an intensive care setting, with 70 patients having been colonised or infected, linked to reusable axillary temperature probes, indicating environmental persistence and healthcare-associated transmission [38]. Despite a bundle of infection control interventions, the incidence of new cases was reduced only after removal of the temperature probes. All outbreak sequences formed a single genetic cluster within the *C. auris* South African clade (III), most likely due to a single undetected introduction in 2013.

### *C. auris* and COVID-19

Early in the pandemic, patients with severe COVID-19 infection, including those in intensive care, were particularly vulnerable to bacterial and fungal co-infection [42,44]. With studies having independently identified that underlying respiratory illness and mechanical ventilation in intensive care are significant predictors of *C. auris* colonisation and infection, it was highlighted that the SARS-CoV-2 pandemic could provide conditions for widespread novel outbreaks of *C. auris* [23,45].

Although the spread of resistant pathogens, including *C. auris*, has been observed amongst critically ill COVID-19 patients during peaks of the pandemic in certain countries, including Italy and the USA, this has not been the experience of all areas [46,47]. In the UK, despite large numbers of patients in intensive care settings, *C. auris* outbreaks were not identified during the pandemic.

#### The Americas

The Pan American Health Organization (PAHO) released an epidemiological alert in 2021 in response to *C. auris* outbreaks during the COVID-19 pandemic [48]. In 2020, seven countries had documented cases of *C. auris*, mostly in patients with COVID-19, including Brazil, Guatemala, Mexico, Peru, the USA, Panama, and Colombia; *C. auris* had not been identified in four of these countries prior to the pandemic. Several outbreaks of *C. auris* have been identified in the USA in COVID-19 units of acute care hospitals, and new cases without links to known cases or healthcare abroad have also been identified across multiple states [47,49]. In Brazil, two colonised patients were identified in a COVID-19 intensive care unit in December 2020 [50]. Screening was undertaken and an outbreak was subsequently identified with 8/47 (17 %) patients testing positive from the axilla, as well as positive findings from environmental screening. Digital thermometers demonstrated the highest rate of positive cultures from environmental screening, followed by bed rails, vital sign monitors/intravenous infusion pumps, and tray tables [50]. With travel restrictions during the pandemic and the absence of travel history among the colonised patients, it was hypothesised that the species was introduced several months before recognition of the first case [51].

In Guatemala, two isolates were detected on the same surgical unit, the first isolated in soft tissue and bone biopsies in a patient with osteomyelitis and the second from a surgical site infection, with neither case being COVID-19 related [48]. A hospital outbreak in Mexico also started with the identification of infection in a patient without COVID-19; however, it later spread to 12 patients within a COVID-19 intensive care unit, with 3 of 4 intensive care areas affected within 3 months [52]. Mortality was high (5/6; 83.3 %) amongst patients with candidaemia.

In Peru, two patients with respiratory illness, one with latent TB and one with COVID-19 infection, were identified as having *C. auris* [48]. Both had a central venous catheter *in situ*, indwelling urinary catheter, and were on mechanical ventilation. In Panama, a significant association with COVID-19 was identified, with 124 isolates of *C. auris* having been identified prior to the PAHO epidemiological alert, of which 108 were in patients with COVID-19 infection [48]. In Colombia, 340 cases of *C. auris* were identified in 2020, several of which were in patients hospitalised with SARS-CoV-2 [48].

#### Europe

In Spain, *C. auris* became the most isolated *Candida* species from blood cultures after the start of the pandemic, associated with a large outbreak in a tertiary hospital that commenced in 2017 but worsened during the pandemic [53]. This was linked with overoccupancy in the intensive care unit, higher workload of healthcare workers, and poor compliance with infection prevention and control (IPC) measures. In Italy, the index case of *C. auris* was identified in 2019, and a nosocomial outbreak subsequently declared in patients hospitalised in intensive care with COVID-19 infection [46]. A single genetic lineage was identified, suggesting a point source. There was also a high rate of MDR organisms identified in patients with COVID-19 admitted to intensive care, in addition to *C. auris*. Again, concerns regarding IPC practices were raised and several patients required a high level of care, as well as longer hospital stays, and frequent use of broad-spectrum antibiotics [46]. In response to 277 cases associated with this outbreak, spread across at least 8 healthcare facilities in the Italian region of Liguria, and 11 cases in facilities in the neighbouring region of Emilia-Romagna, ECDC conducted a rapid risk assessment for the European Union [54].

#### Asia

In Lebanon, the first cases and subsequent outbreak of *C. auris* occurred in October 2020, with a total of 14 cases identified in critical care units over 13 weeks [55]. Two patients were identified with *C. auris* during their stay in COVID-19 intensive care after being admitted through the emergency department, with environmental contamination of the department remaining a possible source.

In India, an outbreak of *C. auris* was identified in an intensive care unit with COVID-19 patients [43].

This included 10 patients with *C. auris* bloodstream infection and a case fatality rate of 60 % (6/10). Of note, this was an elderly and comorbid cohort with severe COVID-19 infection and the mortality directly attributable to *C. auris* is difficult to determine. Affected patients had been hospitalised in intensive care for prolonged periods of time (range 20–60 days), and all had indwelling invasive devises such as central venous and urinary catheters, and other risk factors such as the need for mechanical ventilation or steroid treatment and underlying chronic diseases, including diabetes mellitus and hypertension. However, four of the patients who died experienced persistent candidaemia despite micafungin therapy, with recurrence of *C. auris* detected in serial blood cultures.

#### Middle East

COVID-19 and *C. auris* co-infection was identified in the United Arab Emirates, where it was hypothesised that the increment in fungal infections was the result of acquisition in the hospital setting, and higher susceptibility of the patients, given treatment with broad-spectrum antibiotics and immunosuppressive therapies [56].

An epidemiological analysis of *C. auris* cases during the pandemic concluded that changes in practice were likely to have been relevant to the spread, including changes in prescribing practices, perceptions of appropriate use of personal protective equipment (PPE), including extended and excessive use, and an increase in agency staffing with varied levels of training and experience in PPE use and care of COVID-19 patients [57].

It has been concluded in several regions that the pandemic created an opportunistic environment for spread of *C. auris*, posing pressures such as overwhelmed intensive care units, suboptimal infection control practices, PPE use, temporary staffing, and inadequate training [55,57, 58]. A previous review of the literature identified 36 cases of co-infection with *C. auris* and COVID-19, highlighting that most cases were males, with an age range of 25–86 years, and candidaemia was the predominant presentation [59]. Date of onset of infection post-admission ranged from 4 to 45 days, and most patients had significant underlying comorbidities. Almost all had an indwelling urinary catheter, intravenous catheters, and were on broad-spectrum antibiotics, and most had received steroids. The mortality rate was 53 % for 30 cases with documented outcomes, although the number of deaths directly attributable to *C. auris* is uncertain [59]. The high case fatality rate reported in several studies around the world is in contrast to the UK experience, where no fatalities directly attributable to *C. auris* have been reported to date. The reasons underlying this difference are poorly described but likely multifactorial, including factors related to the host, the pathogen, and the wider healthcare system.

### Risk factors

Acquisition of *C. auris* is commonly associated with high-risk healthcare settings and particularly high-dependency contexts such as intensive care units, with findings of prospective screening on admission suggesting a high propensity for nosocomial acquisition [39,60]. Patients may be colonised rapidly during their inpatient stay, some within 4 days [60]. A retrospective analysis of 912 cases worldwide found that a higher proportion of men (61.4 %) were affected [13]. A population-based study in Spain also showed that incidence was higher in autumn and amongst the age group of 65–84 years [61].

Identified risk factors predisposing to *C. auris* infection are similar to those for other *Candida* species. These factors include severe underlying disease with immunosuppression, such as HIV and bone marrow transplantation, corticosteroid therapy, neutropenia, malignancy, those with chronic kidney disease or diabetes mellitus, a prolonged stay in ICU, mechanical ventilation, presence of a central-venous catheter or urinary catheter, prolonged exposure to broad-spectrum antibiotic or antifungal use, underlying respiratory illness, vascular surgery, or surgery within the previous 30 days [23,45, 46, 62, 63].

Review of clinical characteristics associated with *C. auris* found that underlying diseases were high, but that kidney disease was the only significant risk factor for mortality in *C. auris*-infected patients [35]. Overall mortality with *C. auris* infection is reported as high in the literature, up to 40–60 % worldwide, possibly due in part to severe underlying conditions in the at-risk populations, the MDR nature of the pathogen, and limited availability of certain antifungal drugs in some countries [22,64, 65]. However, as mentioned above, the extent to which mortality is directly attributable to *C. auris* infection can be difficult to determine, especially in populations with a higher relative mortality in general, such as those in critical care settings.

Mechanical ventilation is an important risk factor. The rate of *C. auris* colonisation in residents of skilled nursing facilities caring for ventilated patients was found to be up to 10 times higher than the occurrence in nursing facilities without ventilator support [66]. Additional risk factors for colonisation in this cohort of residents in a ventilator-capable nursing facility include one or more acute care hospital visits in the prior 6 months (adjusted OR 4.2; 95 % CI, 1.9–9.6), antibiotic treatment with carbapenems in the prior 90 days (aOR 3.5; 95 % CI, 1.6–7.6), and systemic fluconazole in the prior 90 days (aOR 6.0; 95 % CI, 1.6–22.6) [66].

*C. auris* can affect both adult and paediatric populations. Nosocomial infection in children has been reported largely under the age of 1 year and in those with certain medical conditions, including prematurity and malignancy, and in children with risk factors such as intravascular catheters and those receiving parenteral nutrition [67]. Transmission is also possible from a colonised mother to baby during delivery [3]. Clusters of infection have been identified in neonates, with risk of mortality associated with invasive disease [68,70].

### Identification and typing

Identification of *C. auris* has been challenging using conventional laboratory diagnostic techniques [71]. Genetic analyses have established that *C. auris* is closely related to *C. haemulonii* and *Clavispora* (previously *Candida*) *lusitaniae* [34]. Other common misidentifications are described in Table 1. Isolates should therefore be identified to the species level. Options for species identification are available and include matrix-assisted laser desorption/ionisation time-of-flight (MALDI-TOF) mass spectrometry [72,74]. MALDI-TOF can reliably differentiate *C. auris* from other *Candida* species [75,76]. Accuracy of this system is reliant on the spectra for the sample organism being present in the reference database and care being given to an appropriate extraction method.

**Table 1.** Examples of misidentification of *C. auris* and the corresponding identification method used (table adapted from Elbaradei *et al*. [167] and Kaur *et al*. [63]). Previous yeast names are in parentheses (see [168])

Species misidentification	Method	Reference
*Candida albicans*	MicroScan	[97]
	Vitek-MS	[169]
*Diutina* (*Candida*) *catenulata*	BD Phoenix	[76]
	MicroScan	[76]
*Debaryomyces hansenii* (previously *Candida famata*)	Vitek-2 (software upgrade to version 8.01 includes *C. auris*. It is, however, recommended to confirm isolates identified as *C. haemulonii* and *C. duobushaemulonii, C. famata*, and *C. auris* by MALDI-TOF or DNA sequencing)	[24,136]
	MicroScan	[76]
*Meyerozyma* (*Candida*) *guilliermondii*	MicroScan	[76]
*Candida haemulonii*	BD Phoenix	[76]
	Vitek-2 (software upgrade to version 8.01 includes *C. auris*. It is, however, recommended to confirm isolates identified as *C. haemulonii* and *C. duobushaemulonii, C. famata*, and *C. auris* by MALDI-TOF or DNA sequencing)	[19,24, 75, 76, 136, 170]
	Vitek-MS	[171]
*Clavispora* (*Candida*) *lusitaniae*	MicroScan	[76]
	Vitek-MS	[171]
*Candida parapsilosis*	MicroScan	[76]
	RapID Yeast Plus	[172]
*Candida sake*	Api 20C AUX	[24,171]
*Candida tropicalis*	MicroScan	[97]
*Rhodotorula glutinis*	Api 20C AUX	[19,76]
*Candida duobushaemulonii*	Vitek-2 (software upgrade to version 8.01 includes *C. auris*. It is, however, recommended to confirm isolates identified as *C. haemulonii* and *C. duobushaemulonii, D. hansenii*, and *C. auris* by MALDI-TOF or DNA sequencing)	[136]
*Lachancea* (*Saccharomyces*) *kluyveri*	API ID32C	[173]

**Fig. 2.**
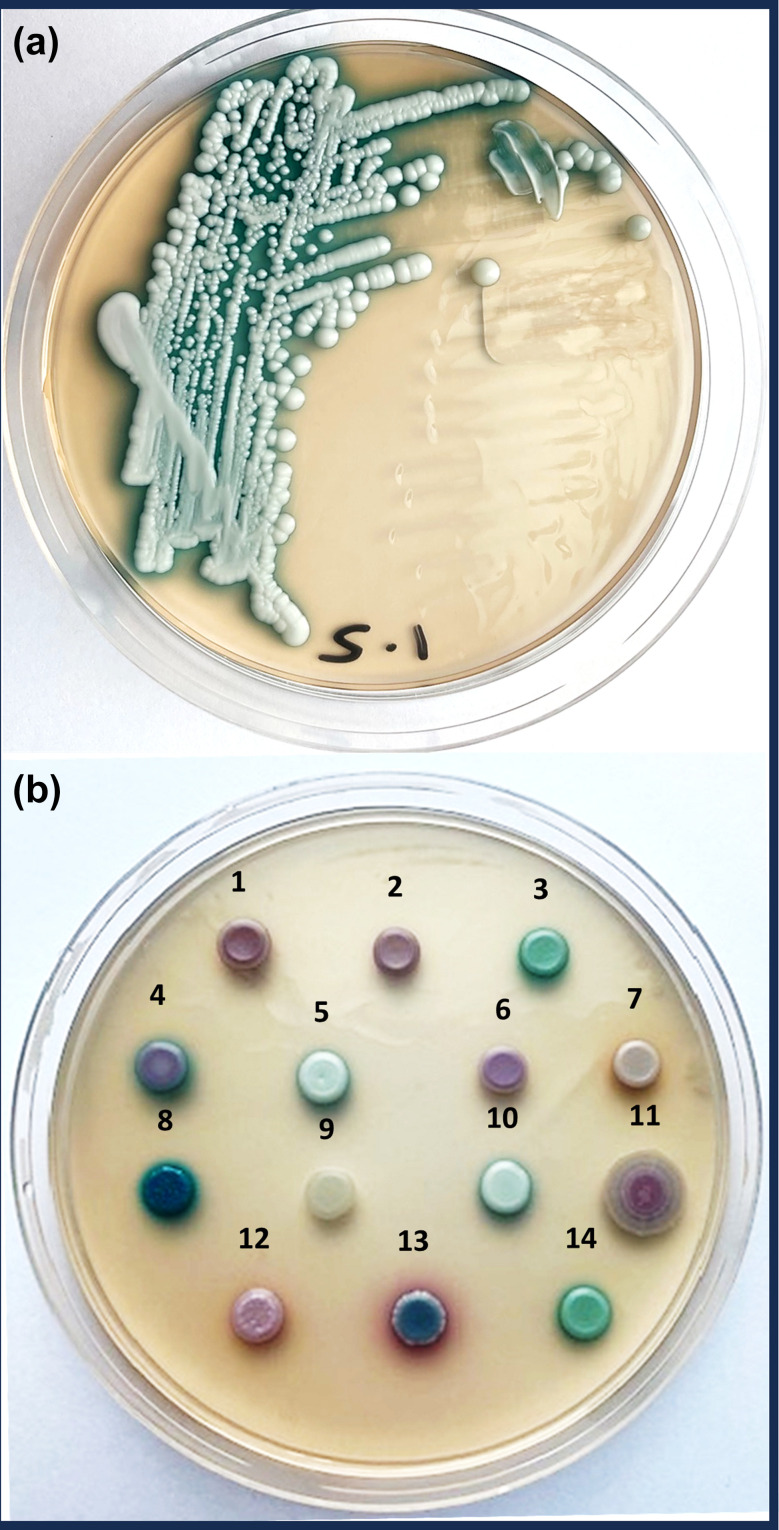
(a) *Candida auris* (clade II) isolate streaked onto a plate of Chromagar Candida Plus with distinctive pale cream colony and diffusing blue halo. (b) Comparative colonial appearances of common species of *Candida* and allied yeast genera spotted onto Chromagar Candida Plus. Clinical isolates tested were: 1*, Nakaseomyces glabratus* (ex-*Candida glabrata*); 2*, Saccharomyces cerevisiae*; 3*, Candida albicans*; 4, *Candida parapsilosis*; 5, *Candida auris* (clade II); 6, *Clavispora lusitaniae* (ex-*Candida lusitaniae*); 7, *Meyerozyma guilliermondii* (ex-*Candida guilliermondii*); 8, *Trichosporon asahii*; 9, *Pichia cactophila* (ex-*Candida inconspicua*); 10, *Candida auris* (clade III); 11, *Pichia kudriavzevii* (ex-*Candida krusei*); 12, *Meyerozyma caribbica* (ex-*Candida fermentati*); 13, *Candida tropicalis*; 14, *Candida dubliniensis*. Plates incubated for 36 h at 35 °C. Image provided by Andrew M. Borman.

A novel chromogenic agar, CHROMagar Candida Plus, appears promising in the identification and differentiation of *C. auris* from other *Candida* species, with *C. auris* colonies appearing as a light blue colour with a blue halo (Fig. 2), and obtaining a sensitivity and specificity of 100 % at 36 h incubation [3,74, 77, 78]. This is a promising alternative to conventional mycological media for the screening of patients who may be colonised or infected with *C. auris*. It has been reported that some strains of *C. parapsilosis* have consistent colonial morphology with *C. auris* on CHROMagar Candida Plus [79]. Therefore, it is recommended that full identification of suspect isolates is performed to confirm an identification of *C. auris* from CHROMagar Candida Plus.

The detection of *C. auris* has been enhanced by the development of molecular tests [80]. Turnaround times and diagnostic sensitivity are improved using molecular technologies, particularly for specimens with a high pre-test probability of *C. auris* colonisation or infection [3]. Molecular identification can be performed by sequencing various DNA loci in specific domains of ribosomal genes (18S rDNA, 28S rDNA or internal transcribed spacers ITS1, ITS2), by conventional or real-time polymerase chain reaction (PCR) and loop-mediated isothermal amplification (LAMP) [24,34].

The identification of *C. auris* using traditional phenotypic and biochemical methods is challenging, and these have largely been superseded by proteomics and molecular methods [3]. Commercial biochemical identification systems commonly used in clinical microbiology laboratories may be unreliable for *C. auris* identification if their system has not been updated to include *C. auris*, due to phenotypic similarities with other species [76,81]. Checking the system for *C. auris* detection ability is therefore required.

Molecular typing of *C. auris* can be performed using a variety of methods, including sequencing of rDNA loci (D1/D2 or ITS regions) to differentiate between the major phylogeographical clades [82]. Further delineation for outbreak analysis requires higher resolution methods, which can be performed by WGS analysis and typing by amplified fragment length polymorphism (AFLP) [3, 34]. International networks are being established to improve genomic capacity and develop analysis pipelines that will support ability to detect outbreaks, identify introductions, and characterise transmission of fungal infections [83,84].

In low-resource settings, the accurate identification of *C. auris* using the methods described above may not be readily achievable. A cross-sectional study exploring the current state of clinical mycology in Africa surveyed 40 institutions across 21 countries and found that few laboratories reported having the capacity to correctly identify *C. auris* [17]. One alternative approach that takes advantage of the salt and thermal tolerance of *C. auris* involves a selective modified Sabouraud agar; when supplemented with 10 % NaCl and dulcitol instead of dextrose as a carbon source and cultured for 72 h at 42 °C, the tentative identification of *C. auris* is possible [85,86]. However, even with a tentative diagnosis of *C. auris,* access to effective therapy is a further challenge. Although sustained advocacy by organisations such as Global Action For Fungal Infections (GAFFI) has raised the profile of fungal infections and supported the addition of echinocandins to the WHO essential medicines list [87], in 2021 the survey by Driemeyer *et al*. [17] found that only 5–22.5 % of respondents from African institutions reported having access to an echinocandin. Since their inclusion in the WHO essential medicines list availability is increasing [88,89], but there are still substantial gaps. An emphasis on the sustainable development of laboratory capacity and workforce in many low-resource settings around the world is essential; not just for fungal infections such as *C. auris* but across the spectrum of pathogens to support antimicrobial stewardship initiatives; enhance the surveillance of antimicrobial resistant infections at local, regional, and national levels; and most importantly improve patient outcomes.

### Antifungal resistance

*C. auris* shows high minimum inhibitory concentrations (MICs) for various antifungal agents. Fluconazole resistance is widespread but not universal; however, this is generally not a therapeutic option for most clinical cases [90]. Additionally, many *C. auris* isolates also show high MICs for newer azoles such as voriconazole, although this varies in a clade-specific manner [91], likely indicating the ineffectiveness of these agents for clinical cases [90]. Clinical cut-off values have, however, not yet been described.

In a collection of *C. auris* isolates (*n*=54) from five continents, 41 % were found to be resistant to two or more antifungal classes [31]. Resistance to echinocandins remains uncommon; however, with widespread use of the drug as first-line therapy, it has been increasingly identified [92]. Pan-resistant strains have also been described [12] and, worryingly, evidence of their transmission between healthcare facilities is emerging [93].

Antifungal drug resistance is thought to be an acquired as well as a shared trait, with the potential to develop over time when fungi are exposed to antifungals [94,95]. The emergence of echinocandin resistance, detected by WGS and phenotypically, within an ongoing prolonged outbreak is concerning [96]. *C. auris* has an intrinsic ability to exhibit or develop resistance very rapidly even while the patient is still undergoing treatment, which is why it is essential to use the antifungals at the right time and at the correct dosage [34]. Antifungal susceptibility testing is therefore recommended for all *C. auris* isolates.

### Clinical characteristics and complications

The clinical spectrum associated with *C. auris* ranges from asymptomatic colonisation to invasive candidiasis, most commonly as healthcare-associated candidaemia [71]. Other healthcare-associated infections reported include intravascular catheter infection, urinary tract and respiratory infection, meningitis, osteomyelitis, surgical site infection, and otomastoiditis [39,92, 97,99]. *C. auris* colonises the axillae, groin, nares, respiratory, and urinary tract of hospitalised patients [6,100]. Intestinal colonisation occurs less frequently but has been associated with *C. auris* urinary infections [101]. Colonisation reportedly results in invasive infections in up to 5–10 % of individuals, with mechanical ventilation and placement of invasive devices identified as two major risk factors [6,102]. The proportion developing invasive infection may vary according to underlying patient risk factors, IPC measures, diagnostic capabilities, and institutional familiarity with *C. auris* outbreaks.

The ability for rapid colonisation of skin and high transmissibility within the healthcare setting can result in prolonged and serious outbreaks [38,39]. Transmission can occur via contact with contaminated items or from affected individuals. Screening of the patient environment has yielded *C. auris* isolates with identical fingerprinting patterns to patient isolates, suggesting shedding of *C. auris* by colonised patients [6]. Contaminated equipment identified during outbreak investigations has included thermometers, temperature probes, pulse oximeters, blood-pressure cuffs, a cloth lanyard, and a call bell [50,51, 103, 104]. The duration of patient colonisation remains uncertain; however, a recent study demonstrated that most patients with *C. auris* colonisation did not have detectable *C. auris* 12 months after discharge to the community setting [21].

Invasive disease can be associated with high mortality in patients within an intensive care setting [63], although the proportion of deaths directly attributable to *C. auris* is difficult to determine. Indeed, this has not been the experience in all settings (e.g. the UK), as described above. Infection can be complicated by multidrug resistance, with bloodstream infections being more difficult to treat, and risks of complications, including spondylodiscitis, endocarditis, and ventriculitis, and clinical syndromes including otomycosis and otomastoiditis [2,99, 105]. Involvement of skin, respiratory, urogenital, and abdominal sites has also been reported [106], including vulvovaginal candidiasis in the context of immunosuppression [107].

Different clades are associated with different clinical presentations and appear to display differences in virulence in animal models [108,109]. A comparison study of *in vivo* pathogenicity of the four initial *C. auris* clades in a neutropenic bloodstream infection murine model found that the highest overall mortality was observed for the South American clade (96 %), followed by the South Asian (80 %), South African (45 %), and East Asian (44 %) clades [109]. The most virulent isolates appear to exhibit pathogenicity comparable to that of *Candida albicans*. In an immunocompetent mouse model, *C. auris* was demonstrated to be highly virulent, but less virulent than *C. albicans* [110].

In relation to patterns of infection, South Asian, and South American clades are commonly associated with bloodstream infections (47 –76 %), whereas the South African clade is associated with a higher percentage of urinary tract infections or colonisation (38 %) [111]. Isolates from the East Asian clade are associated with otitis externa, which also show higher genetic diversity compared to other clades and may indicate an older natural population [111]. All except for the East Asian clade (and more recently discovered Iranian and sixth clades) have been linked to outbreaks causing invasive infections [63].

Bloodstream infections can be followed by complications such as infective endocarditis and spondylodiscitis. Recurrence has also been reported [105]. Case fatality associated with invasive infection is variably reported. In the USA, among patients with blood isolates of *C. auris*, 30 day mortality was reported as 39 % and 90 day mortality as 58 % [112]. In a UK outbreak, however, no mortality was directly attributed to *C. auris* infection [39].

### Treatment considerations

Antifungal agents commonly used to treat *Candida* infections include fluconazole and the echinocandins. *C. auris* isolates are commonly resistant to fluconazole; however, resistance to the other antifungal agents is variable.

Therapeutic recommendations for *C. auris* include echinocandin monotherapy as empirical treatment prior to the results of susceptibility testing, considering common resistance patterns identified to date [34,113, 114]. Although reports of echinocandin- and pan-resistant isolates are increasing, in regions where most strains continue to remain susceptible it is reasonable for echinocandins to remain as first-line treatment [114]. Patients should, however, be monitored for clinical improvement, with follow-up cultures and susceptibility testing, as the organism can develop resistance quickly, including during treatment [115]. If the urinary tract or CNS is involved, dual therapy may be necessary, as some antifungal classes (including echinocandins) do not have bioavailability in either urine or the CNS.

Evidence is lacking about the most appropriate therapy for pan-resistant strains, where resistance to all three major classes of antifungals has been identified (echinocandins, amphotericin B, and azoles) [116]. There is *in vivo* evidence of inhibition of pan-resistant strains through combinations of two antifungal drugs using fixed concentrations, with an effective response noted from flucytosine combinations with amphotericin B, azoles, or echinocandins [117]. There is additional *in vitro* evidence supporting combination therapy against *C. auris* with caspofungin in combination with posaconazole [118] or anidulafungin in combination with manogepix (the active moiety of fosmanogepix) or flucytosine [119]. A novel echinocandin, rezafungin, appears to be promising from *in vitro* studies, including in subsets of echinocandin-resistant *C. auris*, and this is currently undergoing phase 3 trials [120,121]. Fosmanogepix, a first-in-class antifungal with a novel mechanism of action available in intravenous and oral formulations, has shown potential both *in vitro* and in phase 2 studies [122,123]. Both persistent and recurrent *C. auris* bloodstream infections have been documented; animal studies and *in vitro* investigations suggest that micafungin-based combination therapies are promising in this context [113,124, 125].

### Infection prevention and control

*C. auris* is able to grow at higher temperatures than many other fungi and is able to tolerate high salt concentrations [6]. These are important characteristics in its ability to persist in the environment and to survive for long periods of time, creating opportunities for colonisation and transmission. There are additional challenges associated with rapid acquisition and prolonged colonisation, particularly in nosocomial settings, leading to an increased risk of contamination and transmission for weeks or months following exposure [67,92].

Whole-genome sequencing analyses of isolates from patients and their environment have shown that *C. auris* can contaminate surfaces at varying distances from the patient’s bed, including items not frequently touched and those distant from the patient [126,127]. Outbreak investigations have isolated the organism from environmental samples, including those taken from a mattress, bedside table, bed rail, chair, window sill, and from the air [39]; from bed surfaces and equipment such as ventilators, a temperature probe, ECG leads [60]; pulse oximeters [38]; and the floor, patient bedside trollies, air conditioners, a bed sheet, pillow, mobile phone, oxygen mask, and an intravenous pole [8]. Biomedical products and equipment should therefore be single use; reusable equipment should ideally be single-patient use and left in the patient’s room, to ensure thorough terminal decontamination at the time of discharge [128].

Infection prevention and control measures are crucial to reducing transmission. Many of the major world health organisations have published guidance and recommendations regarding the isolation of patients, contact precautions, and cleaning of equipment and environments in contact with *C. auris*, including the UK, the USA, Europe, South Africa, Australia, and Canada (Table S1 [ 36,48, 114, 129,135]).

#### Identification of *C. auris* and screening

*C. auris* can be transmissible whether an individual is infected or colonised, and thus infection control precautions are the same [136]. Screening is widely recommended where transmission has occurred, or where there are close contacts of confirmed *C. auris* cases [3]. Active screening of exposed and potentially exposed patients followed by strict infection control measures has been successful in outbreak management [137]. Evidence suggests that frequently encountered sites for colonisation and therefore for screening include the axilla and groin [60,112]. Screening sites also shown to culture positive for *C. auris*, and that may be relevant, include the rectum, pharynx, urine, nose, mouth, ear, vascular catheter-exit site, and wounds [105,112, 128]. A recent study has demonstrated that adding nasal swabs to composite axilla and groin swabs may yield additional positive isolates, with 25 % of positive findings being on nasal culture alone [112].

The benefits of routine screening of healthcare workers remain unclear; however, screening in outbreak investigations has identified some positive findings [39,126]. *C. auris* has been found on the skin, hands, and nares of care providers and healthcare workers, and screening may therefore be initiated if risk factors are identified [3,138, 139].

The duration of colonisation with *C. auris* remains unknown, although it can be protracted whilst patients remain in healthcare settings [136]. Screening methodologies may not identify all sites of colonisation and deisolation of contacts therefore remains challenging in this setting. An outbreak investigation reported having deisolated close contacts after three consecutive negative screens, whereupon one contact subsequently tested positive again and so weekly screening until discharge was introduced [39]. In response to high rates of detection in New York, a pilot case management programme for people colonised with *C. auris* who were discharged to a community setting was implemented. Although long-term colonisation was observed in some, serial *C. auris* assessments found that approximately two-thirds of patients colonised as inpatients and discharged to a community setting did not remain colonised indefinitely [140]. The time taken for patients to become serially negative was 8.6 months (IQR 5.7–10.8) and for patients who became serially negative, the median time to the first negative was 4.7 months (IQR 3.5–7.5).

#### Transmission-based precautions

Given the evidence around transmission risk associated with *C. auris*, patients colonised or infected should be cared for in a single room with contact precautions, preferably with their own bathroom. A flagging system indicating the isolation should also be visible at the entry of the room and patients should have an alert on their medical records to ensure appropriate isolation on transfer and readmission [134]. Verbal and written communication of infection transmission risk is essential for inter-facility patient transfer, including to community healthcare settings. If single room occupancy is not possible, patients with *C. auris* should as a minimum be cohorted, ideally with single-patient-use commodes and single-use equipment. The importance of hand hygiene should be reinforced with healthcare workers. Hand washing with soap and water, alcohol-based, or chlorhexidine-based hand rub, have all been shown to be effective in eliminating *C. auris* from hands [60,90]. Transmission-based precautions are recommended, including personal protective equipment (PPE) and single-use items, for the duration of the stay in a healthcare facility [136]. PPE including gloves and a long-sleeved gown is recommended for use in contacts with patients with *C. auris* or their environment [26,128, 135].

Special precautions are advised when cleaning or exposed to body fluids in *C. auris-*affected areas. The CDC recommend transmission-based precautions and enhanced barrier precautions that are similar to those for other MDR organisms [135]. Ideally, patients should only be moved for necessary medical procedures and should be last on the list to allow for a thorough clean following, with minimum staff involved [136].

Effective contact precautions are especially important in the context of invasive lines in high-risk cohorts, due to the ability of fungal cells to adhere to the device, and their growth occurring in the form of a biofilm [63].

#### Disinfection of environmental surfaces

*C. auris* can resist certain disinfectants and is well adapted to nosocomial environments, having the potential to remain viable on vertical surfaces in the immediate environment of colonised patients for up to 28 days [60,86]. Additionally, the organism has been cultured from bedding for up to 7 days [60]. *C. auris* can colonise and persist on surfaces for longer than *C. albicans*, and has shown a prolonged metabolic activity [141]. Furthermore, the minimum time taken to acquire *C. auris* from a patient or their immediate environment is 4 h or less, further reinforcing the importance of rigorous IPC measures [39].

Evidence supporting effective products and methods for disinfection of environmental surfaces contaminated by *C. auris* remains limited due to challenges with comparison of studies, with many applying different experimental techniques, the results of which also cannot readily be compared or directly translated to efficacy in real-world scenarios.

Chlorine-based disinfectants, in the form of sodium hypochlorite (NaOCl) and sodium dichloroisocyanurate, are commonly used in the healthcare setting for disinfection, especially for MDR organisms, and these have been the most investigated in relation to *C. auris* [142]. A chlorine-based disinfectant was noted to be effective during an outbreak in the UK in 2015 (1000 p.p.m. Chlor-Clean, Guest Medical), having been used in the daily routine disinfection of the patient area and equipment, with a 10 000 p.p.m. chlorine-based product (Haz-Tab, Guest Medical) having been used for terminal cleaning followed by further disinfection with hydrogen peroxide vapour [39]. *In vitro* studies have also investigated the efficacy of chlorine-based products. A chlorine-based disinfectant (Haz-Tab 1000 p.p.m. chlorine) was tested against different clinical isolates of *C. auris* with an exposure time of 5 min and all isolates had at least a 4.5 log^10^ reduction in growth [143]. A further study evaluated chlorine-based products at 1000 p.p.m. (Chlor-Clean) and 10 000 p.p.m. (Haz-Tab) against clinical isolates of *C. auris* and other *Candida* species, finding that *C. auris* isolates were effectively killed at all concentrations with a minimum of 3 min contact time [144].

Similar studies also concluded that sodium hypochlorite (NaOCl) with concentrations of 1000 p.p.m. or higher were effective in eradicating *C. auris* [128,135, 145]. In relation to effectiveness on surfaces, following application of 1 and 2 % NaOCl to four different surfaces (stainless steel, ceramic, plastic, and glass) for a 10 min contact time, complete eradication of *C. auris* was reported on all surfaces [60]. A further study then investigated the efficacy of NaOCl at 1000 and 10 000 p.p.m. on cellulose matrix, stainless steel, and polyester surfaces inoculated with clinical isolates of *C. auris* [145]. At all concentrations, NaOCl demonstrated significant killing on all substrates at contact times of 5 and 10 min; however, among all the materials tested, complete eradication was only achieved on cellulose substrates. Several commercially available products containing NaOCl have also been found to be ineffective against dry biofilms containing *C. auris* [146].

The efficacy of peracetic acid has been tested on stainless steel, polymer, and cellulose surfaces. Peracetic acid at 2000 p.p.m. was found to have significant killing activity against *C. auris*; however, as with NaOCl, complete eradication was achieved on cellulose matrix but not with steel or plastic [145]. As *C. auris* can survive on plastic surfaces, for efficient removal it has been recommended that peracetic acid be added to NaOCl, peracetic acid (3500 p.p.m.) and sodium dichloroisocyanurate (1000 p.p.m.) having been effective against dry biofilms containing *C. auris* [146].

There are some data regarding the effectiveness of hydrogen peroxide in disinfectant and vaporised form, but with lower levels of supportive evidence [144,147]. Application of hydrogen peroxide vapour appeared to be promising for environmental decontamination in an *in vitro* study, based on a hospital outbreak [144,148]. This is currently recommended as a potential additional safety measure for manual cleaning and disinfection regimes, rather than replacing other regimes [149]. A study of 0.5 and 1.4 % hydrogen peroxide solutions found that they were also effective [147].

Quaternary ammonium compounds are commonly used disinfectants in healthcare settings, but the overall evidence regarding their efficacy for *C. auris* is conflicting, and their use is therefore currently discouraged [150,151].

Further studies highlighted that application in accordance with recommended concentrations and contact times was essential for efficacy with glutaraldehyde, phenols, hydrogen peroxide, and ethanol [60,150]. Two per cent glutaraldehyde and 5 % phenol were found to be effective on multiple surfaces when the recommended contact times of 20 and 60 min, respectively, were used [60]. Alcohol (29.4 % Purell Healthcare Surface Disinfectant) did show some killing activity but was not as effective as chlorine-based disinfectants or hydrogen peroxide [147].

The evidence regarding the effectiveness of ultraviolet-C (UV-C) light is conflicting [150,152]. Several UV surface distinction devices have been tested for efficacy against *C. auris*. These UV systems vary significantly, and each requires individual verification. Reports that found UV to be effective typically quote log reductions between 2.48 and 5.5 for cycle times between 10 and 20 min [153]. This will vary depending on the UV emitter being tested, the distance between the emitter and the contaminated surface, the presence or absence of additional soil, exposure time, the angle of incident radiation, and the degree of shadowing. Other studies have concluded that *C. auris* is significantly less susceptible to killing by UV-C in comparison to other *Candida* species [154], with one study stating the *C. auris* is not effectively killed by standard UV-C disinfection [155] and other studies highlighting strain variability [148,156]. Data are limited and the parameters for effective disinfection with UV-C are not yet well understood.

The CDC currently recommends use of Environmental Protection Agency (EPA)-registered hospital-grade disinfectants that are effective against *Clostridium difficile* spores; primarily chlorine-based products [135]. This has been supported by outbreak management success with stopping transmission [157] and by further investigation of chlorine-based disinfection, concluding effectiveness with appropriate application [143]. If EPA-registered hospital-grade disinfectants are unavailable, suggested alternatives include hydrogen peroxide 0.5–1.4 %, or quaternary ammonium compounds supplemented with isopropyl alcohol and/or ethyl alcohol. The ECDC recommends terminal cleaning using disinfectants and methods with certified antifungal activity, including chlorine-based disinfectants (at a concentration of 1000 p.p.m.), hydrogen peroxide or others. The Public Health Agency of Canada (PHAC) and South African Centre for Opportunistic, Tropical and Hospital Infections (COTHI) interim recommendations include ‘regular’ and terminal cleanings with a chlorine-releasing agent at 1000 p.p.m., and COTHI suggests the addition of hydrogen peroxide vapour, when feasible. The Pan American Health Organization/World Health Organization (PAHO/WHO) recommends cleaning with soap and water followed by disinfection with 0.1 % bleach. PAHO/WHO recommend ‘high activity’ compounds, such as sodium hypochlorite, hydrogen peroxide (and vaporised), and peracetic acid. The Australian Society of Infectious diseases recommends the use of products that claim to have sporicidal activity for disinfection (e.g., ≥1000 p.p.m. bleach, peracetic acid or accelerated hydrogen peroxide).

Terminal cleaning and disinfection of the environment remain an essential element of the IPC precautions required to prevent transmission of *C. auris* from infected or colonised patients. Whilst antimicrobial surfaces and coating will never replace environmental decontamination, this is an area of active research. One example of this is a fast-acting, permanent antimicrobial surface made of compressed sodium chloride (CSC). Pilot data indicate at least 99 % reduction of *C. auris* in 1 min. There are *in vitro* data suggesting that *C. auris* strains can be killed on contact when exposed to caspofungin that is reformulated as a covalently bound surface layer on glass and plastic [158]; however, the risk of selecting resistant strains is uncertain and an important concern.

#### Patient decolonisation

Several studies have investigated products for decolonisation in relation to *C. auris*, with effectiveness having been demonstrated for chlorhexidine gluconate, povidone-iodine, and alcohol [39,60, 143]. The benefits of decolonisation and subsequent recolonisation risk remain unclear. There are therefore no protocols for decolonisation of patients with *C. auris*; an international expert working group were unable to make recommendations due to a lack of evidence [128].

Chlorhexidine gluconate is widely used in healthcare for handwashing, pre-procedure skin preparation, intravascular catheter exit-site care, oral care for prevention of ventilator-associated pneumonia, and whole-body bathing [159]. It is effective for the decolonisation of *Candida* species, although there is little evidence specific to *C. auris* [142]. One study demonstrated that chlorhexidine gluconate (<0.02 % with a contact time of 24 h) was effective in inhibiting growth of planktonic cells and biofilms of clinical isolates of *C. auris* [160]. With concentrations between 0.125 and 1.5 %, and a 3 min contact time, growth of *C. auris* was inhibited and increased efficacy was seen at 3 and 30 h [144]. However, *C. auris* had consistently higher minimal inhibition concentrations (MICs) when compared to other *Candida* species tested. A study of 2 % chlorhexidine gluconate used alone failed to eliminate *C. auris* with a contact time of 2 min. The addition of 70 % isopropyl alcohol (IPA), however, did reduce all six strains of *C. auris* to undetectable levels within 2 min, suggesting that a chlorhexidine/IPA disinfectant could reduce colonisation if applied appropriately [143]. This may cause skin damage, and further evidence is required.

Patients can remain colonised despite daily chlorhexidine washes [39,60]. Decolonisation may, however, reduce *C. auris* bioburden on the skin surface of affected individuals, potentially reducing the risk of transmission [39,92]. The evidence suggests challenges result from persistent colonisation. It remains uncertain if this relates to reinfection from the environment or reduced susceptibility to chlorhexidine. In one study, two patients with persistent colonisation in the groin suffered from diarrhoea, which may have contributed to persistence [60].

In a facility where *C. auris* was endemic, integrating microbial genomic and epidemiological data revealed occult *C. auris* colonisation of multiple body sites not commonly targeted for screening [161]. High concentrations of chlorhexidine were associated with suppression of *C. auris* growth but not with deleterious perturbation of commensal microbes. A murine model study observed that *C. auris* can enter the dermis without causing overt histopathological signs of inflammation, expanding into deeper tissues, which could make decolonisation challenging [162]. This could also explain the reoccurrence of *C. auris* in patients who had serial negative swabs. Interestingly, chlorhexidine antiseptic protected against colonisation of *C. auris* on mouse skin.

A study investigating the *in vitro* yeasticidal activity of povidone-iodine against *C. auris*, compared to *C. albicans* and *Candida glabrata*, was encouraging. The growth of all clinical *C. auris* isolates was inhibited at concentrations between 0.07 and 1.25 %, which is below many of the commercially available concentration of 10 %, with a minimum contact time of 3 min [144].

A murine skin colonisation model was used to test fungal burden reduction following treatment with 1 % terbinafine or 1 % clotrimazole in a proprietary Advanced Penetration Technology formulation. This found that both treatments significantly reduced fungal burden compared to that in control groups [163]. Compounds with antimicrobial activity have been assessed for efficacy in antifungal decolonisation, including triclosan, boric acid, and zinc oxide, which can be used for long periods of time without an abrasive skin effect. Antifungal activity of boric acid and triclosan was demonstrated against multiple *Candida* species, including a clade of *C. auris* [164].

## Concluding remarks

*Candida auris* is increasingly detected at widespread geographical locations, and prevalence is likely to be greater than what is currently reported. Detection has improved in many countries known to be affected by *C. auris*. The organism should be considered when unidentified or unusual *Candida* species are isolated from patients who fail to respond to empirical antifungal therapy. Colonisation and infection have been primarily detected in high-dependency settings to date, and important risk factors include intravascular catheters and mechanical ventilation. The COVID-19 pandemic therefore had an extremely detrimental impact in several countries worldwide, with widespread transmission and *C. auris* co-infection frequently associated with overcrowding, inadequate PPE use, and suboptimal IPC practice. This was also associated with high mortality, although that directly attributable to *C. auris* cannot be established. Accurately evaluating prevalence outside of high-dependency settings has been a challenge and requires further study to inform guidance.

It is clearly difficult to completely prevent transmission following identification of someone colonised with *C. auris*, even whilst employing strict IPC and isolation techniques, but control has been demonstrated [137,165, 166].

Although treatment of asymptomatic colonisation is not currently widely recommended in the literature, this requires further review. Potential benefit to individuals and implications for wider transmission has been suggested; however, efficacy is uncertain. The duration patients can remain colonised, and thus how long patients should be isolated after first detection, is also uncertain. Continuing isolation for the duration of an inpatient stay has been recommended, given the risk of transmission. Although the CDC recommend deisolation following two consecutive negative screening swabs, this criterion is uncommonly met and recolonisation is possible.

Antifungal susceptibility testing should be performed on all isolates, and empirical treatment with an echinocandin is recommended whilst awaiting results. The development of increasingly resistant strains is, however, a concern. Future strategies include combination therapies and novel and alternative treatments, such as natural compounds (e.g. rocaglates), photodynamic therapy, and novel triazoles (e.g. PC945), but these require further evaluation. Antimicrobial stewardship plays a key role in limiting unnecessary antimicrobial use and ensuring appropriate case management.

A multidisciplinary response is needed to control *C. auris* transmission in a healthcare setting and should emphasise outbreak preparedness and response, rapid contact tracing and isolation, cohorting of patients and staff, hand hygiene, strict IPC, dedicated or single-use equipment, appropriate disinfection, transfers, and discharge.

Despite intensive research efforts over the past decade, several unanswered questions regarding *C. auris* remain [18] Box 1.

Box 1. **Priority research questions**

Epidemiology

- What is the global burden of disease associated with and attributable to *Candida auris*?
- Can wastewater sequencing support *Candida auris* surveillance?
- Is there an ecological niche and is this relevant for human transmission?

Pathogenesis

- How does the skin microbiome influence *Candida auris* colonisation, and vice versa?

Antimicrobial resistance

- What are the optimal clinical breakpoints for *Candida auris*?

Diagnosis

- What strategies best support the implementation of fungal diagnostics in resource-constrained settings?
- Can point-of-care rapid diagnostics reliably inform outbreak investigations?

Treatment

- What is the most effective treatment for *Candida auris* infections at sites that are poorly penetrated by echinocandins (e.g. urinary tract, central nervous system)?
- When should combination therapy be used and which agents?

Infection prevention and control

- Why does *Candida auris* colonisation persist for so long?
- Is *Candida auris* colonisation any different from that of other MDR pathogens, such as MRSA, ESBLs, CPEs?
- Which virulence factor(s) determine the ability of *Candida auris* to persist in the healthcare environment?
- What is the optimal disinfection regimen for daily and terminal cleaning?
- How can we improve the design of multi-use equipment and the hospital environment to facilitate cleaning and reduce the environmental burden of infectious pathogens?

**Box 1 Priority research questions**

## supplementary material

10.1099/jmm.0.001820 Table S1.

10.1099/jmm.0.001820 Supplementary Material 1.

## Abbreviations

AFLP: amplified fragment length polymorphism
CDC: Centers for Disease Control and Prevention (USA)
CINAHL: Cumulative Index to Nursing & Allied Health
CNS: central nervous system
COTHI: South African Centre for Opportunistic, Tropical and Hospital Infections
COVID-19: Coronavirus SARS-CoV-2 virus
CSC: compressed sodium chloride
DNA: deoxyribonucleic acid
ECDC: European Centre for Disease Control
ECG: electrocardiogram
EPA: Environmental Protection Agency
GAFFI: Global Action For Fungal Infections
HIV: Human Immunodeficiency Virus
ICU: intensive care unit
IPA: isopropyl alcohol
IPC: infection prevention and control
ITS: internal transcribed spacers
LAMP: loop-mediated isothermal amplification
MALDI-TOF: matrix-assisted laser desorption/ionisation time-of-flight
MDR: multi-drug resistant
MIC: minimum inhibitory concentration
NICE: National Institute for Health and Care Excellence
PAHO: Pan American Health Organisation
PCR: polymerase chain reaction
PHAC: Public Health Agency of Canada
PPE: personal protective equipment
SNPs: single nucleotide polymorphisms
UK: United Kingdom
UKHSA: United Kingdom Health Security Agency
USA: United States of America
WGS: whole genome sequencing
WHO: World Health Organisation

## References

[1] Satoh K, Makimura K, Hasumi Y, Nishiyama Y, Uchida K (2009). *Candida auris* sp. nov., a novel ascomycetous yeast isolated from the external ear canal of an inpatient in a Japanese hospital. Microbiol Immunol.

[2] Kim M-N, Shin JH, Sung H, Lee K, Kim E-C (2009). *Candida haemulonii* and closely related species at 5 university hospitals in Korea: identification, antifungal susceptibility, and clinical features. Clin Infect Dis.

[3] Keighley C, Garnham K, Harch SAJ, Robertson M, Chaw K (2021). *Candida auris:* diagnostic challenges and emerging opportunities for the clinical microbiology laboratory. Curr Fungal Infect Rep.

[4] Fisher MC, Denning DW (2023). The WHO fungal priority pathogens list as a game-changer. Nat Rev Microbiol.

[5] Khatamzas E, Madder H, Jeffery K (2019). Neurosurgical device-associated infections due to *Candida auris* - three cases from a single tertiary center. J Infect.

[6] Ahmad S, Alfouzan W (2021). *Candida auris*: epidemiology, diagnosis, pathogenesis, antifungal susceptibility, and infection control measures to combat the spread of infections in healthcare facilities. Microorganisms.

[7] Tanne JH (2021). *Candida auris*: CDC warns of risk of new, drug resistant strain in US. BMJ.

[8] Yadav A, Singh A, Wang Y, Haren MHV, Singh A (2021). Colonisation and transmission dynamics of *Candida auris* among chronic respiratory diseases patients hospitalised in a chest hospital. J Fungi.

[9] Chybowska AD, Childers DS, Farrer RA (2020). Nine things genomics can tell us about *Candida auris*. Front Genet.

[10] Chayaporn S, Karrie Kwan Ki K, Kar Mun L, Mei Gie T, Patipan B (2023). Discovery of the sixth *Candida auris* clade in Singapore. medRxiv.

[11] Price TK, Mirasol R, Ward KW, Dayo AJ, Hilt EE (2021). Genomic characterizations of Clade III lineage of *Candida auris*, California, USA. Emerg Infect Dis.

[12] Lyman M, Forsberg K, Reuben J, Dang T, Free R (2021). Notes from the field: transmission of pan-resistant and echinocandin-resistant *Candida auris* in health care facilities - Texas and the District of Columbia, January-April 2021. MMWR Morb Mortal Wkly Rep.

[13] Kohlenberg A, Monnet DL, Plachouras D, Candida auris survey collaborative group, Candida auris survey collaborative group includes the following national experts (2022). Increasing number of cases and outbreaks caused by *Candida auris* in the EU/EEA, 2020 to 2021. Euro Surveill.

[14] Nelson R (2023). Emergence of resistant *Candida auris*. Lancet Microbe.

[15] van Schalkwyk E, Mpembe RS, Thomas J, Shuping L, Ismail H (2019). Epidemiologic shift in Candidemia driven by *Candida auris*, South Africa. Emerg Infect Dis.

[16] Chakrabarti A, Sood P, Rudramurthy SM, Chen S, Kaur H (2015). Incidence, characteristics and outcome of ICU-acquired candidemia in India. Intensive Care Med.

[17] Driemeyer C, Falci DR, Oladele RO, Bongomin F, Ocansey BK (2022). The current state of clinical mycology in Africa: a European Confederation of Medical Mycology and International Society for Human and Animal Mycology survey. Lancet Microbe.

[18] Jeffery-Smith A, Taori SK, Schelenz S, Jeffery K, Johnson EM (2018). *Candida auris*: a review of the literature. Clin Microbiol Rev.

[19] Lee WG, Shin JH, Uh Y, Kang MG, Kim SH (2011). First three reported cases of nosocomial fungemia caused by *Candida auris*. J Clin Microbiol.

[20] Oh BJ, Shin JH, Kim M-N, Sung H, Lee K (2011). Biofilm formation and genotyping of *Candida haemulonii*, *Candida pseudohaemulonii*, and a proposed new species (*Candida auris*) isolates from Korea. Med Mycol.

[21] Desnos-Ollivier M, Fekkar A, Bretagne S (2021). Earliest case of *Candida auris* infection imported in 2007 in Europe from India prior to the 2009 description in Japan. J Mycol Med.

[22] Lamoth F, Kontoyiannis DP (2018). The *Candida auris* alert: facts and perspectives. J Infect Dis.

[23] Borman AM, Johnson EM (2020). *Candida auris* in the UK: introduction, dissemination, and control. PLoS Pathog.

[24] Chowdhary A, Sharma C, Duggal S, Agarwal K, Prakash A (2013). New clonal strain of *Candida auris*, Delhi, India. Emerg Infect Dis.

[25] CDC Tracking Candida auris: Centers for Disease Prevention and Control 2021. https://www.cdc.gov/fungal/candida-auris/tracking-c-auris.html.

[26] ECDC (2018). Rapid risk assessment: *Candida auris* in healthcare settings - Europe. Stockholm:European Centre for Disease Prevention and Control (ECDC). https://www.ecdc.europa.eu/en/publications-data/rapid-risk-assessment-candida-auris-healthcare-settings-europe.

[27] Plachouras D, Lötsch F, Kohlenberg A, Monnet DL, Candida auris survey collaborative group (2020). *Candida auris*: epidemiological situation, laboratory capacity and preparedness in the European Union and European Economic Area*, January 2018 to May 2019. Euro Surveill.

[28] Mathur P, Hasan F, Singh PK, Malhotra R, Walia K (2018). Five-year profile of candidaemia at an Indian trauma centre: high rates of *Candida auris* blood stream infections. Mycoses.

[29] Sharp A, Muller-Pebody B, Charlett A, Patel B, Gorton R (2021). Screening for *Candida auris* in patients admitted to eight intensive care units in England, 2017 to 2018. Euro Surveill.

[30] Forsberg K, Woodworth K, Walters M, Berkow EL, Jackson B (2019). *Candida auris*: the recent emergence of a multidrug-resistant fungal pathogen. Med Mycol Open Access.

[31] Lockhart SR, Etienne KA, Vallabhaneni S, Farooqi J, Chowdhary A (2017). Simultaneous emergence of multidrug-resistant *Candida auris* on 3 continents confirmed by whole-genome sequencing and epidemiological analyses. Clin Infect Dis.

[32] Chow NA, de Groot T, Badali H, Abastabar M, Chiller TM (2019). Potential fifth clade of *Candida auris*, Iran, 2018. Emerg Infect Dis.

[33] Armaki MT, Omran SM, Jafarzadeh J, Tavakoli M, Kiakojuri K (2021). First fluconazole-resistant *Candida auris* isolated from fungal Otitis in Iran. Curr Med Mycol.

[34] Jeffery-Smith A, Taori SK, Schelenz S, Jeffery K, Johnson EM (2018). *Candida auris*: a review of the literature. Clin Microbiol Rev.

[35] Hu S, Zhu F, Jiang W, Wang Y, Quan Y (2021). Retrospective analysis of the clinical characteristics of *Candida auris* infection worldwide from 2009 to 2020. Front Microbiol.

[36] UKHSA (2024). *Candida auris*: laboratory investigation, management and infection prevention andcontrol: UK Health Security Agency. https://www.gov.uk/government/publications/candida-auris-laboratory-investigation-management-and-infection-prevention-and-control.

[37] Brown CS, Guy R (2019). National public health response to *Candida auris* in England. J Fungi.

[38] Eyre DW, Sheppard AE, Madder H, Moir I, Moroney R (2018). A *Candida auris* outbreak and its control in an intensive care setting. N Engl J Med.

[39] Schelenz S, Hagen F, Rhodes JL, Abdolrasouli A, Chowdhary A (2016). First hospital outbreak of the globally emerging *Candida auris* in a European hospital. Antimicrob Resist Infect Control.

[40] Taori SK, Khonyongwa K, Hayden I, Athukorala GDA, Letters A (2019). *Candida auris* outbreak: mortality, interventions and cost of sustaining control. J Infect.

[41] Taori SK, Rhodes J, Khonyongwa K, Szendroi A, Smith M (2022). First experience of implementing *Candida auris* real-time PCR for surveillance in the UK: detection of multiple introductions with two international clades and improved patient outcomes. J Hosp Infect.

[42] Al-Hatmi AMS, Mohsin J, Al-Huraizi A, Khamis F (2021). COVID-19 associated invasive candidiasis. J Infect.

[43] Chowdhary A, Tarai B, Singh A, Sharma A (2020). Multidrug-resistant *Candida auris* infections in critically Ill coronavirus disease patients, India, April-July 2020. Emerg Infect Dis.

[44] Lansbury L, Lim B, Baskaran V, Lim WS (2020). Co-infections in people with COVID-19: a systematic review and meta-analysis. J Infect.

[45] Rudramurthy SM, Chakrabarti A, Paul RA, Sood P, Kaur H (2017). *Candida auris* candidaemia in Indian ICUs: analysis of risk factors. J Antimicrob Chemother.

[46] Magnasco L, Mikulska M, Giacobbe DR, Taramasso L, Vena A (2021). Spread of Carbapenem-resistant Gram-negatives and *Candida auris* during the COVID-19 pandemic in critically Ill patients: one step back in antimicrobial stewardship?. Microorganisms.

[47] Prestel C, Anderson E, Forsberg K, Lyman M, de Perio MA (2021). *Candida auris* outbreak in a COVID-19 specialty care unit - Florida, July-August 2020. MMWR Morb Mortal Wkly Rep.

[48] PAHO (2021). Epidemiological alert: *Candida auris* outbreaks in health care services in the contextof the COVID-19 pandemic: Pan American Health Organisation (PAHO). https://iris.paho.org/bitstream/handle/10665.2/53377/EpiUpdate6February2021_eng.pdf?sequence=1&isAllowed=y.

[49] CDC (2020). Fungal diseases and COVID-19: Centers for Disease Prevention and Control. https://www.cdc.gov/fungal/covid-fungal.html.

[50] Nobrega de Almeida J, Brandão IB, Francisco EC, de Almeida SLR, de Oliveira Dias P (2021). Axillary digital thermometers uplifted a multidrug-susceptible *Candida auris* outbreak among COVID-19 patients in Brazil. Mycoses.

[51] de Almeida JN, Francisco EC, Hagen F, Brandão IB, Pereira FM (2021). Emergence of *Candida auris* in Brazil in a COVID-19 intensive care unit. J Fungi.

[52] Villanueva-Lozano H, Treviño-Rangel R de J, González GM, Ramírez-Elizondo MT, Lara-Medrano R (2021). Outbreak of *Candida auris* infection in a COVID-19 hospital in Mexico. Clin Microbiol Infect.

[53] Mulet Bayona JV, Tormo Palop N, Salvador García C, Fuster Escrivá B, Chanzá Aviñó M (2021). Impact of the SARS-CoV-2 pandemic in Candidaemia, invasive aspergillosis and antifungal consumption in a tertiary hospital. J Fungi.

[54] ECDC (2022). Rapid risk assessment: *Candida auris* outbreak in healthcare facilities in northern Italy, 2019-2021.

[55] Allaw F, Kara Zahreddine N, Ibrahim A, Tannous J, Taleb H (2021). First *Candida auris* outbreak during a COVID-19 pandemic in a tertiary-care center in Lebanon. Pathogens.

[56] Senok A, Alfaresi M, Khansaheb H, Nassar R, Hachim M (2021). Coinfections in patients hospitalized with COVID-19: a descriptive study from the United Arab Emirates. Infect Drug Resist.

[57] Hanson BM, Dinh AQ, Tran TT, Arenas S, Pronty D (2021). *Candida auris* Invasive Infections during a COVID-19 case surge. Antimicrob Agents Chemother.

[58] Codda G, Di Pilato V, Ball L, Giacobbe DR, Willison E (2021). Molecular epidemiological investigation of a nosocomial cluster of *C. Auris*: evidence of recent emergence in Italy and ease of transmission during the COVID-19 pandemic. J Fungi.

[59] Goravey W, Ali GA, Ali M, Ibrahim EB, Al Maslamani M (2021). Ominous combination: COVID-19 disease and *Candida auris* fungemia-case report and review of the literature. Clin Case Rep.

[60] Biswal M, Rudramurthy SM, Jain N, Shamanth AS, Sharma D (2017). Controlling a possible outbreak of *Candida auris* infection: lessons learnt from multiple interventions. J Hosp Infect.

[61] Ruiz-Azcona L, Santibañez M, Roig FJ, Vanaclocha H, Ventero MP (2021). Isolation of *Candida auris* in large hospitals in the autonomous community of Valencia; population-based study (2013-2017). Rev Iberoam Micol.

[62] Briano F, Magnasco L, Sepulcri C, Dettori S, Dentone C (2022). *Candida auris* Candidemia in critically ill, colonized patients: cumulative incidence and risk factors. Infect Dis Ther.

[63] Kaur H, Wadhwa K, Jain K, Yadav A (2021). Multidrug-resistant *Candida auris*: a global challenge. J Appl Biol Biotechnol.

[64] Al-Rashdi A, Al-Maani A, Al-Wahaibi A, Alqayoudhi A, Al-Jardani A (2021). Characteristics, risk factors, and survival analysis of *Candida auris* cases: results of one-year National Surveillance Data from Oman. J Fungi.

[65] Shaukat A, Al Ansari N, Al Wali W, Karic E, El Madhoun I (2021). Experience of treating *Candida auris* cases at a general hospital in the state of Qatar. IDCases.

[66] Rossow J, Ostrowsky B, Adams E, Greenko J, McDonald R (2021). Factors associated with *Candida auris* colonization and transmission in skilled nursing facilities with ventilator units, New York, 2016-2018. Clin Infect Dis.

[67] Berrio I, Caceres DH, Coronell R W, Salcedo S, Mora L (2021). Bloodstream infections with *Candida auris* among children in Colombia: clinical characteristics and outcomes of 34 cases. J Pediatric Infect Dis Soc.

[68] Alvarado-Socarras JL, Vargas-Soler JA, Franco-Paredes C, Villegas-Lamus KC, Rojas-Torres JP (2021). A cluster of neonatal infections caused by *Candida auris* at a large referral center in Colombia. J Pediatric Infect Dis Soc.

[69] Mesini A, Saffioti C, Mariani M, Florio A, Medici C (2021). First case of *Candida auris* colonization in a preterm, extremely low-birth-weight newborn after vaginal delivery. J Fungi.

[70] Ramya GM, Balakrishnan U, Chandrasekaran A, Abiramalatha T, Amboiram P (2021). Candida auris, an emerging pathogen - challenge in the survival of microprimies. Indian J Med Microbiol.

[71] Chowdhary A, Sharma C, Meis JF (2017). *Candida auris*: a rapidly emerging cause of hospital-acquired multidrug-resistant fungal infections globally. PLoS Pathog.

[72] Fasciana T, Cortegiani A, Ippolito M, Giarratano A, Di Quattro O (2020). *Candida auris*: an overview of how to screen, detect, test and control this emerging pathogen. Antibiotics.

[73] Lockhart SR, Lyman MM, Sexton DJ (2022). Tools for detecting a “Superbug”: updates on *Candida auris* testing. J Clin Microbiol.

[74] Borman AM, Fraser M, Johnson EM (2021). CHROMagar^TM^ Candida Plus: a novel chromogenic agar that permits the rapid identification of *Candida auris*. Med Mycol Open Access.

[75] Kathuria S, Singh PK, Sharma C, Prakash A, Masih A (2015). Multidrug-resistant *Candida auris* misidentified as *Candida haemulonii*: characterization by matrix-assisted laser desorption ionization-time of flight mass spectrometry and DNA sequencing and its antifungal susceptibility profile Variability by Vitek 2, CLSI broth microdilution, and Etest method. J Clin Microbiol.

[76] Mizusawa M, Miller H, Green R, Lee R, Durante M (2017). Can multidrug-resistant *Candida auris* be reliably identified in clinical microbiology laboratories?. J Clin Microbiol.

[77] Mulet Bayona JV, Salvador García C, Tormo Palop N, Gimeno Cardona C (2020). Candida for the detection of *Candida auris* in surveillance samples. Diagn Microbiol Infect Dis.

[78] Mulet Bayona JV, Salvador Garcia C, Tormo Palop N, Valentin Martin A, Gonzalez Padron C (2022). Plus for detection of *Candida auris* and other *Candida* species from surveillance and environmental samples: a multicenter study. J Fungi.

[79] Sasoni N, Maidana M, Latorre-Rapela MG, Morales-Lopez S, Berrio I (2022). *Candida auris* and some *Candida parapsilosis* strains exhibit similar characteristics on CHROMagarTMCandida plus. Med Mycol.

[80] White PL, Price JS, Cordey A, Backx M (2021). Molecular diagnosis of yeast infections. Curr Fungal Infect Rep.

[81] Chatterjee S, Alampalli SV, Nageshan RK, Chettiar ST, Joshi S (2015). Draft genome of a commonly misdiagnosed multidrug resistant pathogen *Candida auris*. BMC Genom.

[82] Borman AM, Szekely A, Johnson EM (2017). Isolates of the emerging pathogen *Candida auris* present in the UK have several geographic origins. Med Mycol.

[83] Bagal UR, Phan J, Welsh RM, Misas E, Wagner D (2022). MycoSNP: a portable workflow for performing whole-genome sequencing analysis of *Candida auris*. Methods Mol Biol.

[84] Chow N, Parnell L, Escandon P, Govender N, Chiller T (2022). S5.5a standing up FungiNet: genomic epidemiology and surveillance of fungal diseases. Med Mycol Open Access.

[85] González-Durán E, Contreras-Pérez CU, Caceres DH, Ríos-Rosas C, Piñón-Ortega J de J (2022). The use of readily available laboratory tests for the identification of the emerging yeast *Candida auris* in Mexico. Arch Microbiol.

[86] Welsh RM, Bentz ML, Shams A, Houston H, Lyons A (2017). Survival, persistence, and isolation of the emerging multidrug-resistant pathogenic yeast *Candida auris* on a plastic health care surface. J Clin Microbiol.

[87] WHO (2023). WHO model list of essential medicines - 23rd list, 2023: World Health Organisation. https://www.who.int/publications/i/item/WHO-MHP-HPS-EML-2023.02.

[88] GAFFI (2023). GAFFI antifungal drug maps - micafungin: global action for fungal infections. https://drugsmaps.azurewebsites.net/maps/map/micafungin.

[89] Kneale M, Bartholomew JS, Davies E, Denning DW (2016). Global access to antifungal therapy and its variable cost. J Antimicrob Chemother.

[90] Aldejohann AM, Wiese-Posselt M, Gastmeier P, Kurzai O (2022). Expert recommendations for prevention and management of *Candida auris* transmission. Mycoses.

[91] Szekely A, Borman AM, Johnson EM (2019). *Candida auris* Isolates of the southern Asian and South African lineages exhibit different phenotypic and antifungal susceptibility profiles *in vitro*. J Clin Microbiol.

[92] Vallabhaneni S, Kallen A, Tsay S, Chow N, Welsh R (2017). Investigation of the first seven reported cases of *Candida auris*, a globally emerging invasive, multidrug-resistant fungus-United States, May 2013-August 2016. Am J Transplant.

[93] Dang T, Bassett J, Souri A, Nutt A, Honza H (2022). Transmission of pan-resistant and echinocandin-resistant *Candida auris* between healthcare facilities during the COVID-19 pandemic. Am J Infect Control.

[94] Lockhart SR, Berkow EL, Chow N, Welsh RM (2017). *Candida auris* for the clinical microbiology laboratory: not your Grandfather’s *Candida* species. Clin Microbiol Newslett.

[95] Gupta AK, Venkataraman M (2022). Antifungal resistance in superficial mycoses. J Dermatolog Treat.

[96] Codda G, Willison E, Magnasco L, Morici P, Giacobbe DR (2023). In vivo evolution to echinocandin resistance and increasing clonal heterogeneity in *Candida auris* during a difficult-to-control hospital outbreak, Italy, 2019 to 2022. Euro Surveill.

[97] Morales-López SE, Parra-Giraldo CM, Ceballos-Garzón A, Martínez HP, Rodríguez GJ (2017). Invasive infections with multidrug-resistant yeast *Candida auris*, Colombia. Emerg Infect Dis.

[98] Chowdhary A, Anil Kumar V, Sharma C, Prakash A, Agarwal K (2014). Multidrug-resistant endemic clonal strain of *Candida auris* in India. Eur J Clin Microbiol Infect Dis.

[99] Choi HI, An J, Hwang JJ, Moon SY, Son JS (2017). Otomastoiditis caused by *Candida auris*: case report and literature review. Mycoses.

[100] Chakrabarti A, Sood P (2021). On the emergence, spread and resistance of *Candida auris*: host, pathogen and environmental tipping points. J Med Microbiol.

[101] Piatti G, Sartini M, Cusato C, Schito AM (2022). Colonization by *Candida auris* in critically ill patients: role of cutaneous and rectal localization during an outbreak. J Hosp Infect.

[102] Southwick K, Ostrowsky B, Greenko J, Adams E, Lutterloh E (2022). A description of the first *Candida auris*-colonized individuals in New York State, 2016-2017. Am J Infect Control.

[103] Patterson CA, Wyncoll D, Patel A, Ceesay Y, Newsholme W (2021). Cloth lanyards as a source of intermittent transmission of *Candida auris* on an ICU. Crit Care Med.

[104] Decker BK, Clancy CJ (2021). Lanyards as source of a *Candida auris* outbreak: as you investigate the environment, do not overlook hand hygiene. Crit Care Med.

[105] Ruiz-Gaitán A, Moret AM, Tasias-Pitarch M, Aleixandre-López AI, Martínez-Morel H (2018). An outbreak due to *Candida auris* with prolonged colonisation and candidaemia in a tertiary care European hospital. Mycoses.

[106] Schwartz IS, Smith SW, Dingle TC (2018). Something wicked this way comes: what health care providers need to know about *Candida Auris*. Canada communicable disease report = Releve des maladies Transmissibles au Canada.

[107] Krishnasamy L, Senthilganesh J, Saikumar C, Nithyanand P (2021). Biofilm-forming fluconazole-resistant *Candida auris* causing vulvovaginal candidiasis in an immunocompetent patient: a case report. Asian Pac J Trop Med.

[108] Borman AM, Szekely A, Johnson EM (2016). Comparative pathogenicity of United Kingdom isolates of the emerging pathogen *Candida auris* and other key pathogenic *Candida* species. mSphere.

[109] Forgács L, Borman AM, Prépost E, Tóth Z, Kardos G (2020). Comparison of *in vivo* pathogenicity of four *Candida auris* clades in a neutropenic bloodstream infection murine model. Emerg Microbes Infect.

[110] Fakhim H, Vaezi A, Dannaoui E, Chowdhary A, Nasiry D (2018). Comparative virulence of *Candida auris* with *Candida haemulonii*, *Candida glabrata* and *Candida albicans* in a murine model. Mycoses.

[111] Tian S, Bing J, Chu Y, Chen J, Cheng S (2021). Genomic epidemiology of *Candida auris* in a general hospital in Shenyang, China: a three-year surveillance study. Emerg Microbes Infect.

[112] Adams E, Quinn M, Tsay S, Poirot E, Chaturvedi S (2013). *Candida auris* in Healthcare facilities.

[113] Lepak AJ, Zhao M, Berkow EL, Lockhart SR, Andes DR (2017). Pharmacodynamic optimization for treatment of invasive *Candida auris* infection. Antimicrob Agents Chemother.

[114] CDC Treatment and management of infections and colonization: centers for disease prevention and control 2021. https://www.cdc.gov/fungal/candida-auris/c-auris-treatment.html.

[115] Biagi MJ, Wiederhold NP, Gibas C, Wickes BL, Lozano V (2019). Development of high-level echinocandin resistance in a patient with recurrent *Candida auris* Candidemia secondary to chronic Candiduria. Open Forum Infect Dis.

[116] CDC. Clinical alert to U.S (2016). Healthcare facilities: centers for disease prevention and control. https://www.cdc.gov/fungal/candida-auris/candida-auris-alert.html?CDC_AA_refVal=https%3A%2F%2Fwww.cdc.gov%2Ffungal%2Fdiseases%2Fcandidiasis%2Fcandida-auris-alert.html.

[117] O’Brien B, Liang J, Chaturvedi S, Jacobs JL, Chaturvedi V (2020). Pan-resistant *Candida auris*: New York subcluster susceptible to antifungal combinations. Lancet Microbe.

[118] Balla N, Kovács F, Balázs B, Borman AM, Bozó A (2022). Synergistic interaction of caspofungin combined with posaconazole against *FKS* wild-type and mutant *Candida auris* planktonic cells and biofilms. Antibiotics.

[119] John LLH, Thomson DD, Bicanic T, Hoenigl M, Brown AJP (2023). Heightened efficacy of anidulafungin when used in combination with manogepix or 5-flucytosine against *Candida auris In Vitro*. Antimicrob Agents Chemother.

[120] Tóth Z, Forgács L, Locke JB, Kardos G, Nagy F (2019). In vitro activity of rezafungin against common and rare *Candida* species and *Saccharomyces cerevisiae*. J Antimicrob Chemother.

[121] Ham YY, Lewis JS, Thompson GR (2021). Rezafungin: a novel antifungal for the treatment of invasive candidiasis. Future Microbiol.

[122] Pfaller MA, Huband MD, Rhomberg PR, Bien PA, Castanheira M (2022). Activities of manogepix and comparators against 1,435 recent fungal isolates collected during an International Surveillance Program (2020). Antimicrob Agents Chemother.

[123] Vazquez JA, Pappas PG, Boffard K, Paruk F, Bien PA (2023). Clinical efficacy and safety of a novel antifungal, fosmanogepix, in patients with Candidemia caused by *Candida auris:* results from a phase 2 trial. Antimicrob Agents Chemother.

[124] Fakhim H, Chowdhary A, Prakash A, Vaezi A, Dannaoui E (2017). In vitro interactions of echinocandins with triazoles against multidrug-resistant *Candida auris*. Antimicrob Agents Chemother.

[125] Jaggavarapu S, Burd EM, Weiss DS (2020). Micafungin and amphotericin B synergy against *Candida auris*. Lancet Microbe.

[126] Escandón P, Chow NA, Caceres DH, Gade L, Berkow EL (2019). Molecular epidemiology of *Candida auris* in Colombia reveals a highly related, countrywide colonization with regional patterns in amphotericin B resistance. Clin Infect Dis.

[127] Pacilli M, Kerins JL, Clegg WJ, Walblay KA, Adil H (2020). Regional emergence of *Candida auris* in Chicago and lessons learned from intensive follow-up at 1 ventilator-capable skilled nursing facility. Clin Infect Dis.

[128] Kenters N, Kiernan M, Chowdhary A, Denning DW, Pemán J (2019). Control of *Candida auris* in healthcare institutions: outcome of an International Society for antimicrobial chemotherapy expert meeting. Int J Antimicrob Agents.

[129] ECDC (2016). *Candida auris* in healthcare settings: European Centre for Disease Prevention and Control. https://www.ecdc.europa.eu/en/publications-data/candida-auris-healthcare-settings.

[130] COTHI (2016). Interim guidance for management of *Candida auris* infections in South African hospitals: centre for opportunistic, tropical and hospital infections. https://www.nicd.ac.za/assets/files/2016-12-22%20InterimNICDRecommdtnsCAuris.pdf.

[131] Govender NP, Avenant T, Brink A, Chibabhai V, Cleghorn J (2019). Federation of Infectious Diseases Societies of Southern Africa guideline: recommendations for the detection, management and prevention of healthcare-associated *Candida auris* colonisation and disease in South Africa. S Afr J Infect Dis.

[132] Ontario Agency for Health Protection and Promotion (Public Health Ontario) (2019). Provincial infectious diseases advisory committee interim guide for infection prevention and control of *Candida auris*. https://www.publichealthontario.ca/en/About/External-Advisory-Committees/PIDAC-IPC.

[133] Vuichard-Gysin D, Sommerstein R, Martischang R, Harbarth S, Kuster SP (2020). *Candida auris* - recommendations on infection prevention and control measures in Switzerland. Swiss Med Wkly.

[134] Ong CW, Chen SC-A, Clark JE, Halliday CL, Kidd SE (2019). Diagnosis, management and prevention of *Candida auris* in hospitals: position statement of the Australasian Society for Infectious Diseases. Intern Med J.

[135] CDC Infection prevention and control for *Candida auris*: centers for disease prevention and control 2021. https://www.cdc.gov/fungal/candida-auris/c-auris-infection-control.html.

[136] Caceres DH, Forsberg K, Welsh RM, Sexton DJ, Lockhart SR (2019). *Candida auris*: a review of recommendations for detection and control in healthcare settings. J Fungi.

[137] Alshamrani MM, El-Saed A, Mohammed A, Alghoribi MF, Al Johani SM (2021). Management of *Candida auris* outbreak in a tertiary-care setting in Saudi Arabia. Infect Control Hosp Epidemiol.

[138] Černáková L, Roudbary M, Brás S, Tafaj S, Rodrigues CF (2021). *Candida auris*: a quick review on identification, current treatments, and challenges. Int J Mol Sci.

[139] Keri VC, Kumar A, Singh G, Xess I, Khan MA (2021). Fungal carriage on healthcare workers’ hands, clothing, stethoscopes and electronic devices during routine patient care: a study from a tertiary care center. J Prev Med Hyg.

[140] Bergeron G, Bloch D, Murray K, Kratz M, Parton H (2021). *Candida auris* colonization after discharge to a community setting: New York City, 2017-2019. Open Forum Infect Dis.

[141] Chaabane F, Graf A, Jequier L, Coste AT (2019). Review on antifungal resistance mechanisms in the emerging pathogen *Candida auris*. Front Microbiol.

[142] Ku TSN, Walraven CJ, Lee SA (2018). *Candida auris*: disinfectants and implications for infection control. Front Microbiol.

[143] Moore G, Schelenz S, Borman AM, Johnson EM, Brown CS (2017). Yeasticidal activity of chemical disinfectants and antiseptics against *Candida auris*. J Hosp Infect.

[144] Abdolrasouli A, Armstrong-James D, Ryan L, Schelenz S (2017). In vitro efficacy of disinfectants utilised for skin decolonisation and environmental decontamination during a hospital outbreak with *Candida auris*. Mycoses.

[145] Kean R, Sherry L, Townsend E, McKloud E, Short B (2018). Surface disinfection challenges for *Candida auris*: an in-vitro study. J Hosp Infect.

[146] Ledwoch K, Maillard JY (2018). *Candida auris* dry surface biofilm (DSB) for disinfectant efficacy testing. Materials.

[147] Cadnum JL, Shaikh AA, Piedrahita CT, Sankar T, Jencson AL (2017). Effectiveness of disinfectants against *Candida auris* and other *Candida* species. Infect Control Hosp Epidemiol.

[148] de Groot T, Chowdhary A, Meis JF, Voss A (2019). Killing of *Candida auris* by UV-C: importance of exposure time and distance. Mycoses.

[149] Janniger EJ, Kapila R (2021). Public health issues with *Candida auris* in COVID-19 patients. World Med Health Policy.

[150] Zatorska B, Moser D, Diab-Elschahawi M, Ebner J, Lusignani LS (2021). The effectiveness of surface disinfectants and a micellic H2O2 based water disinfectant on *Candida auris*. J Med Mycol.

[151] Cadnum JL, Pearlmutter BS, Haq MF, Jencson AL, Donskey CJ (2021). Effectiveness and real-world materials compatibility of a novel hydrogen peroxide disinfectant cleaner. Am J Infect Control.

[152] Pearlmutter BS, Haq MF, Cadnum JL, Jencson AL, Carlisle M (2022). Efficacy of relatively low-cost ultraviolet-C light devices against *Candida auris*. Infect Control Hosp Epidemiol.

[153] Rutala WA, Kanamori H, Gergen MF, Sickbert-Bennett EE, Weber DJ (2022). Inactivation of *Candida auris* and *Candida albicans* by ultraviolet-C. Infect Control Hosp Epidemiol.

[154] Fu L, Le T, Liu Z, Wang L, Guo H (2020). Different efficacies of common disinfection methods against *Candida auris* and other *Candida* species. J Infect Public Health.

[155] Astrid F, Beata Z, Van den Nest M, Julia E, Elisabeth P (2021). The use of a UV-C disinfection robot in the routine cleaning process: a field study in an Academic hospital. Antimicrob Resist Infect Control.

[156] Chatterjee P, Choi H, Ochoa B, Garmon G, Coppin JD (2020). Clade-specific variation in susceptibility of *Candida auris* to broad-spectrum ultraviolet C light (UV-C). Infect Control Hosp Epidemiol.

[157] Kurt AF, Kuskucu MA, Balkan IiI, Baris A, Yazgan Z (2021). *Candida auris* fungemia and a local spread taken under control with infection control measures: first report from Turkey. Indian J Med Microbiol.

[158] Lamont-Friedrich SJ, Kidd SE, Giles C, Griesser HJ, Coad BR (2021). *Candida auris* susceptibility on surfaces coated with the antifungal drug caspofungin. Med Mycol Open Access.

[159] Babiker A, Lutgring JD, Fridkin S, Hayden MK (2021). Assessing the potential for unintended microbial consequences of routine chlorhexidine bathing for prevention of healthcare-associated infections. Clin Infect Dis.

[160] Sherry L, Ramage G, Kean R, Borman A, Johnson EM (2017). Biofilm-forming capability of highly virulent, multidrug-resistant *Candida auris*. Emerg Infect Dis.

[161] Proctor DM, Dangana T, Sexton DJ, Fukuda C, Yelin RD (2021). Integrated genomic, epidemiologic investigation of *Candida auris* skin colonization in a skilled nursing facility. Nat Med.

[162] Huang X, Hurabielle C, Drummond RA, Bouladoux N, Desai JV (2021). Murine model of colonization with fungal pathogen *Candida auris* to explore skin tropism, host risk factors and therapeutic strategies. Cell Host Microbe.

[163] Ghannoum M, Herrada J, McCormick TS, Long L (2023). A novel transdermal application for clearing skin colonization by *Candida auris*. Antimicrob Agents Chemother.

[164] Gavilanes-Martínez MA, Coral-Garzón A, Cáceres DH, García AM (2021). Antifungal activity of boric acid, triclosan and zinc oxide against different clinically relevant *Candida* species. Mycoses.

[165] Reimer-McAtee M, Corsi G, Reed E, Boston KM, Yalamanchili H (2021). Successful implementation of the CDC recommendations during the care of 2 patients with *Candida auris* in in-patient rehabilitation and intensive care settings. Am J Infect Control.

[166] Mohammed A, Alshamrani MM, El-Saed A, Cabanalan H, Alghoribi M (2019). Management of *Candida auris* exposure among patients and healthcare workers at tertiary care setting. Antimicrob Resist Infect Control.

[167] ElBaradei A (2020). A decade after the emergence of Candida auris: what do we know?. Eur J Clin Microbiol Infect Dis.

[168] Borman AM, Johnson EM (2021). Name changes for fungi of medical importance, 2018 to 2019. J Clin Microbiol.

[169] Kim T-H, Kweon OJ, Kim HR, Lee M-K (2016). Identification of uncommon *Candida* species using commercial identification systems. J Microbiol Biotechnol.

[170] Dudiuk C, Berrio I, Leonardelli F, Morales-Lopez S, Theill L (2019). Antifungal activity and killing kinetics of anidulafungin, caspofungin and amphotericin B against *Candida auris*. J Antimicrob Chemother.

[171] Ruiz Gaitán AC, Moret A, López Hontangas JL, Molina JM, Aleixandre López AI (2017). Nosocomial fungemia by *Candida auris*: first four reported cases in continental Europe. Rev Iberoam Micol.

[172] Snayd M, Dias F, Ryan RW, Clout D, Banach DB (2018). Misidentification of *Candida auris* by RapID yeast plus, a commercial, biochemical enzyme-based manual rapid identification system. J Clin Microbiol.

[173] Iguchi S, Itakura Y, Yoshida A, Kamada K, Mizushima R (2019). *Candida auris*: a pathogen difficult to identify, treat, and eradicate and its characteristics in Japanese strains. J Infect Chemother.

