# Supplementary material for: The laboratory investigation, management, and infection prevention and control of Candida auris: a narrative review to inform the 2024 national guidance update in England
Source: J Med Microbiol. 2024 May 21;73(5):001820. doi: 10.1099/jmm.0.001820 (PMC11165919; doi:10.1099/jmm.0.001820)
Supplement: Table S1. [file jmm-73-01820-s001.pdf]

Supplementary table 1: Internationally published guidance and recommendations regarding the isolation of patients, contact precautions, and cleaning of equipment and environments in contact with *C. auris*. Abbreviations: UKHSA – United Kingdom Health Security Agency; LVS – low vaginal swab; PPE – personal protective equipment; ppm – parts per million; MDR – multi-drug resistant; CDC – Centers for Disease Control and Prevention; ECDC – European Centre for Disease Prevention and Control; COTHI – Centre for Opportunistic, Tropical and Hospital Infections; HCW – healthcare worker; UV-C – ultraviolet C; WHO – World Health Organization; PAHO – Pan American Health Organization; COVID-19 – Coronavirus disease 2019; ICU – intensive care unit; FIDSSA - Federation of Infectious Diseases Societies of Southern Africa.

Recommendation(s)						
Body	Patient screening	Contact precautions	Contact screening	Decolonisation	Environmental management	Community management
UKHSA (36)	Recommended in units with ongoing cases or colonisations; those arriving from affected units (UK or abroad); overnight stay in a healthcare facility abroad in the previous year. Screening sites such as groin, axilla, nose, throat, urine, perineal area, rectal area, and stool. Consider screening LVS, sputum, endotracheal secretions, drain fluid, wounds, and cannula. Rescreening of patients known to have been previously colonised. Deisolation of screen-positive patients is not recommended apart from units with experience in managing *C. auris*	Side room with en suite facilities where possible; isolation of all patients from affected UK or international hospital until screening is available; strict adherence to hand hygiene using soap and water, followed by alcohol rub to dry hands; PPE with gloves and aprons, or gowns if there is a high risk of body or body fluid contact; briefing of visitors regarding contact precautions; single-patient-use items such as blood pressure cuffs should be considered; for cleaning *C. auris*-exposed areas, glove and apron use with subsequent appropriate hand decontamination	If there is novel detection in a unit, close contacts should be screened and isolated or cohorted; if this occurs in a critical care or other augmented care setting, do a point prevalence screening survey of the entire unit or ward; if the index patient is isolated, identify all *Candida* species isolates from the same unit to the species level using a method able to detect *C. auris*; review *Candida* spp. detected in the same ward areas in the 4 weeks prior to diagnosis of the index patient in case of unrecognised transmission; deisolation with 3 negative screens >24h apart	Strict adherence to central and peripheral catheter care bundles, urinary catheter care bundle, care of the tracheostomy site; decolonisation is not routinely recommended; skin decolonisation with chlorhexidine washes in critically ill patients and on a case-by-case basis in other augmented care settings can be considered; consider use of mouth gargles with chlorhexidine and use of topical nystatin and terbinafine for topical management of key sites	Use of chlorine-releasing agent at 1,000 ppm for cleaning contact environments; change privacy curtains; for equipment, consider single-use items or discarding less expensive items that are difficult to decontaminate; all equipment should be cleaned in accordance with the manufacturer's instructions; terminal cleaning when patient leaves the environment; schedule affected patients last for theatre/procedures/ imaging; for waste and linen disposal, follow local policy as for other MDR organisms; training and supervision of cleaning staff until competent	Nurse in a single room with en suite facilities when possible; if single room is not possible, the colonised individual should not share a room with an immunocompromised individual; thorough environmental cleaning and disinfection with a chlorine-releasing agent at 1,000 ppm of available chlorine; follow standard infection control precautions; ensure that staff are trained in the use of PPE and hand hygiene; special care should be taken with wound, catheter, and device care

CDC (114, 135)	Patients who have had an overnight stay in a healthcare facility outside the United States in the previous one year especially if they are from a country with documented cases. Strongly consider screening when patients have had such inpatient healthcare exposures outside the United States and have infection or colonisation with carbapenemase-producing Gram-negative bacteria. Axilla and groin screening; additional sites as directed clinically or by previously positive sites; periodic reassessment for presence of colonisation at 1- to 3-month intervals; for deisolation - 2 or more assessments 1 week apart with negative results (off antifungals)	Single room with standard contact precautions; gown and gloves; hand hygiene precautions including Alcohol-based hand sanitiser (ABHS) is effective against *C. auris* and is the preferred method for cleaning hands when not visibly soiled. If visibly soiled, wash with soap and water. Increase hand hygiene audits on units where patients with *C. auris* reside. Consider re-educating healthcare personnel on hand hygiene. Implementation of transmission-based precautions for *C. auris* is like its use for other multidrug-resistant organisms.	At a minimum, screen roommates at healthcare facilities, where the index patient resided in the previous month. Consider also screening patients who require higher levels of care (e.g., mechanical ventilation) and who overlapped on the ward or unit with the index patient for 3 or more days. Consider the patient's prior healthcare exposures and contacts when devising a screening strategy. Strongly consider more extensive screening, such as a point prevalence survey (i.e., every patient on a given unit or floor where transmission is suspected), if there is evidence or suspicion of ongoing transmission in a facility. Screen for colonisation using a composite swab of the patient's bilateral axilla and groin.	CDC does not recommend treatment of *C. auris* identified from non-invasive sites (such as respiratory tract, urine, and skin colonisation) when there is no evidence of infection	Thorough daily and terminal cleaning/disinfection using an Environmental Protection Agency-registered disinfectant effective against C. difficile spores	Do not restrict nursing home residents to rooms and perform hand hygiene; if receiving health input, gown and glove contact precautions; thorough cleaning of shared equipment. Screen nursing home resident contacts if within a month of detection of case. In addition to standard precautions, use alcohol-based hand sanitiser as the preferred method for cleaning hands when not visibly soiled. If hands are visibly soiled, wash with soap and water. Wear disposable gown and gloves when entering the area of house where providing patient care. Gowns and gloves should be removed and disposed of carefully; perform hand hygiene when leaving the patient care area. Ensure reusable equipment is disinfected with an agent effective against *C. auris* before use with another patient.

ECDC (129)	All patients from in-country or internationally affected units transferred in; conduct active surveillance in accordance with specified protocol; screening sites include urine, faeces, wounds, drain fluid, respiratory samples	Contact precautions, single room isolation; patient cohorting; dedicated nursing staff for colonised or infected patients; hand hygiene. Precautions to be applied until discharge from hospital.	Detection of a case should trigger an investigation including detailed case review and screening of close contact patients for carriage. More extensive contact tracing can be considered based on a case-by-case risk assessment. Cross-sectional patient screening in outbreak setting. Screening of close contacts for carriage with axilla and groin swabs. Other sites can be sampled, if clinically relevant or indicated.	Currently no established protocols for decolonisation.	Terminal cleaning and disinfection of rooms using chlorine-based disinfectants (at a concentration of 1,000 ppm), hydrogen-peroxide or other disinfectants with documented fungicidal activity. Quaternary ammonium compound disinfectants should be avoided. Single use equipment or equipment specific to a *C. auris* patient or cohort is preferable. Environmental sampling in outbreak setting.	No specific recommendations
South Africa (COTHI) (130)	Routine screening not advised	Single room with en suite or cohorting of patients; hand hygiene using soap and water or alcohol rub; gloves and aprons for patient contact; adherence to venous and urinary catheter and tracheostomy care bundles; advise visitors regarding importance of hand hygiene and encourage to wear aprons	No specific recommendations	Not recommended	Schedule affected patients last for theatre/ procedures/ imaging; regular cleaning and disinfection with chlorine-releasing agent at 1,000 ppm; terminal cleaning and disinfection of bed space; consider terminal cleaning with hydrogen peroxide vapor; clean multiuse equipment thoroughly; cleaning of all contact areas	No specific recommendations
Australasian Society for Infectious Diseases (134)	Close contacts, patients transferred from facilities with endemic *C. auris* or admitted following stay in overseas healthcare	All patients colonised or infected with *C. auris* should be placed in a single room (with dedicated bathroom or commode or pans) and	Index cases of *C. auris* should prompt screening of close contacts. Initially, screening of ward contacts recommended. In facilities with >1 patient with *C. auris*,	*In vitro* data suggest *C. auris* is susceptible to chlorhexidine, but persistent colonisation is described despite daily chlorhexidine bathing.	Products that claim to have sporicidal activity should be used for disinfection (e.g., ≥1,000 ppm bleach, peracetic acid or accelerated hydrogen peroxide).	No specific recommendations

	institutions should be pre-emptively isolated and screened for colonisation.<br>Composite swabs of the axilla and groin should be collected.	managed using Standard and Contact Precautions for the entire hospital stay and all subsequent hospital admissions.<br>Consider cohorting patients infected or colonised with *C. auris* if single rooms are unavailable.<br>Single-patient use, or single-use equipment should be used wherever possible.<br>If a patient needs to leave their room to go to another department, the receiving department should be notified of the patient's *C. auris* status and advised regarding necessary precautions.	screening activities should be broadened. Composite axilla and groin swabs (as a minimum) are recommended. Close contacts should be pre-emptively isolated and screened for colonisation.<br>Close contacts of a *C. auris* patient (i.e., current room contacts and room contacts within the prior month (including at other wards/facilities)) can be de-isolated after three consecutive negative screens at least 24 h apart.<br>All other persons undergoing screening can be de-isolated after a single negative screen. HCW screening should only be considered if epidemiological investigations suggest HCW are a likely source or where ongoing transmission is identified despite adherence to other interventions	Routine patient decolonisation cannot be definitively recommended but may be considered in settings where transmission persists despite other interventions.	Quaternary ammonium compounds are not reliably effective against *C. auris* and should not be used. Use both detergent and disinfectant as per manufacturer instructions, particularly with respect to contact time. Non-touch disinfection techniques (e.g., UV-C light, hydrogen peroxide vapour) may be used as an adjunct but should not replace the use of a sporicidal chemical agents and must be preceded by environmental cleaning.	
WHO/PAHO (48)	Sampling all patients coming from hospitals where cases of *C. auris* colonisation/infection have been reported.<br>For screening, sampling from the	Isolation of cases in individual rooms. When more than one case is identified, and single rooms are not available, cohort isolation recommended, ensuring	Screen all patients who are in the same hospital ward, especially patients with: confirmed COVID-19; atypical pneumonia; risk factors (diabetes, immunosuppression,	Treatment of *C. auris* colonisation is not recommended, although it is advisable to consider prophylaxis, according to local recommendations, in high-risk colonised	Use a disinfectant effective against *C. auris* at least daily, especially on frequently touched surfaces, including those in close contact with the patient (e.g., chairs, beds, patient tables,	No specific recommendations

	axilla, oropharynx, nostrils, groin, urine, and rectum. If collecting samples from all these sites is not feasible, at least sample from the groin or axilla (pooling sample analysis can be carried out).	that beds are at least one meter apart and standard and transmission-based precautions are followed.	chronic kidney disease, recent surgery; prolonged hospitalisation in ICUs; invasive methods, such as haemodialysis, parenteral feeding, or mechanical ventilation; or use of broad-spectrum antibiotics; and direct case contacts.	patients, prior to surgery or certain invasive procedures	monitors, infusion pumps, cables, keyboards, respirator, among others). Consider the type of surface material to be cleaned and select the best disinfectant. Recommended high activity compounds – sodium hypochlorite, hydrogen peroxide (and vaporised), peracetic acid and hydrogen peroxide. Quaternary ammonium compounds are to be avoided.	
South Africa (FIDSSA Guideline) (131)	Routine screening of all newly admitted patients is not feasible or recommended in a resource-constrained setting.	Isolation, cohorting and use of personal protective equipment such as disposable aprons and gloves.	Screening may be considered in an outbreak situation to establish colonisation of epidemiologically linked patients, defined as currently sharing a cubicle. If in shared rooms with or without semi-permanent barriers, this includes all patients in a shared physical area. Also consider screening any roommates the case had during the last month. Screening for colonisation can be performed by submitting skin swabs from the axilla and groin for selective culture. Screening of healthcare personnel during	No recommendations	Clean daily with a neutral detergent and water and then wipe with sodium-hypochlorite (1,000 ppm) solution. Other disinfectants such as quaternary ammonium compounds and ethyl alcohol should not be used. There is insufficient evidence to recommend routine UV light disinfection though hydrogen peroxide vapour or wipes may be considered.	No specific recommendations

			an outbreak is not routinely recommended.			
Public Health Ontario (132)	Test patients or residents transferred from a facility with recent *C. auris* transmission or with endemic *C. auris*. Consider testing patients or residents admitted to a health care facility outside of Canada within the previous 12 months.	Patients identified as colonised or infected with *C. auris* should be placed into a single room with dedicated toileting facilities (toilet or commode) not shared with other patients or residents; staff and visitors entering the room should use both Routine Practices and Contact Precautions.	When a single case of *C. auris* is identified (unless the case was identified and isolated promptly upon admission), test current and previous roommates and current ward. In an outbreak, test current and previous roommates, all current ward mates and any other patient or resident who may have had a significant exposure based on the epidemiology of the outbreak. The following specimens are recommended at a minimum: a nasal swab plus a combined bilateral axillary and groin swab and other sites as indicated.	No recommendations	Sodium hypochlorite and improved hydrogen peroxide (0.5%, 1.4%) are effective agents against *C. auris* while quaternary ammonium compounds are not. Rooms should be disinfected daily, and single use equipment recommended. Hydrogen peroxide vapour and ultraviolet light may reduce levels of environmental contamination with *C. auris*, however, it is essential that the room is first cleaned and disinfected using standard processes.	No specific recommendations
Switzerland (133)	Screening of high-risk patients transferred from a hospital abroad with recent *C. auris* transmission or with endemic *C. auris*. Screen using composite bilateral swab of axilla and groin and a swab from both nostrils. Other sites as indicated.	Contact precautions and place the patient in a single room with dedicated bathroom/toilet. Reinforce standard precautions especially hand hygiene with an alcohol-based hand rub.	Detection of *C. auris* in a non-isolated patient should trigger screening of close contacts, including those sharing the room or equipment (if uncertain screening all close contacts since admission of the case should be considered). Include all current ward mates if detection in a non-isolated patient during an intensive care stay or if	No recommendations	For small surfaces, use 70% ethanol or alcohol-based disinfectants as per manufacturer directions. For alcohol sensitive or large surfaces, use disinfectant compound with fungicidal activity. Twice daily.	No specific recommendations

			secondary case. Healthcare worker screening not recommended unless substantial evidence as potential source or ongoing transmission			
