# Supplementary material for: The laboratory investigation, management, and infection prevention and control of Candida auris: a narrative review to inform the 2024 national guidance update in England
Source: J Med Microbiol. 2024 May 21;73(5):001820. doi: 10.1099/jmm.0.001820 (PMC11165919; doi:10.1099/jmm.0.001820)
Supplement: Supplementary Material 1. [file jmm-73-01820-s002.pdf]

Supplementary appendix 1 – *Candida auris* global epidemiology

Aim

- To identify countries that have reported cases or outbreaks of *Candida auris*
- To describe countries reporting *C. auris* according to:
  - Detected vs outbreaks vs evidence of endemicity
  - Year of first detection

Methods

- PubMed literature search using free text search term "*Candida auris*"
- Reference lists were reviewed for additional publications not identified through PubMed
- Review articles were included to identify grey literature data; for example, national level surveillance data sets that would not routinely appear in PubMed
- Definitions:
  - Cases detected = cases reported without an association with a healthcare-associated infection (HCAI) outbreak
  - HCAI outbreak = cases reported in association with a HCAI outbreak
  - Endemic = *C. auris* is reported as one of the most common causes of invasive candidiasis; or *C. auris* is reported as being widespread within a country or multiple regions within a country
- Both the year of publication and, where available, the year that the first case was reported in a country were extracted
- World maps were generated using the *rworldmap* package in R (v4.3.1)

Results

To date a total of 61 countries have reported cases or HCAI outbreaks of *C. auris* [1-324]. The table below summarises countries according to whether they have reported detection of cases, HCAI outbreaks, or whether there is evidence that *C. auris* has become endemic within a country (or multiple regions of that country). Some of the *C. auris* detections in returning travellers included travel history and country of probable healthcare exposure, for example Ethiopia and Angola. These have not been included in the dataset.

**Detected**		**HCAI outbreaks**		**Endemic**
Austria	New Zealand	Algeria	Lebanon	India
Bangladesh	Norway	Australia	Mexico	Kenya
Belgium	Panama	Brazil	Nigeria	Kuwait
Chile	Peru	Canada	Oman	South Africa
China	Poland	Colombia	Pakistan	Spain
Costa Rica	Portugal	Denmark	Qatar	United States
Czech Republic	Reunion	France	Romania	
Egypt	Singapore	Germany	Russia	
Finland	South Korea	Greece	Saudi Arabia	
Guatemala	Sudan	Hong Kong	United Arab Emirates	
Iran	Sweden	Israel	United Kingdom	
Ireland	Switzerland	Italy	Venezuela	
Japan	Taiwan			
Jordan	Thailand			
Malaysia	Turkey			
The Netherlands				

Conclusion

The number of countries reporting cases and outbreaks of *C. auris* continues to increase rapidly and it is now present throughout the world. There is evidence that *Candida auris* has become endemic in several countries, often in countries with multiple co-occurring antimicrobial resistant infections. It is highly likely that *C. auris* occurs in countries that have not yet reported a case; laboratory capacity to detect *C. auris* is lacking in many countries, particularly in the African region.

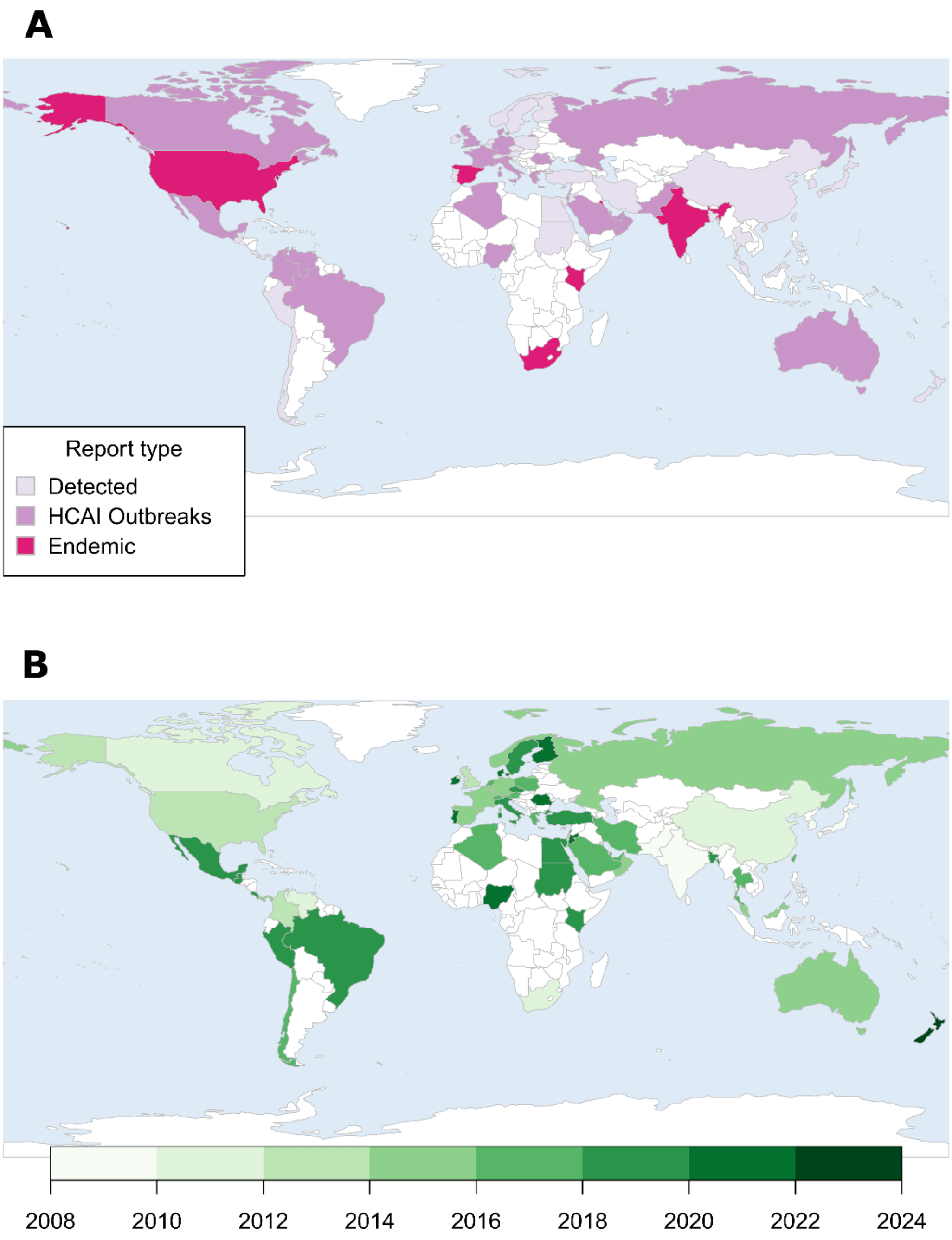

Figure 1: Global epidemiology of *Candida auris*. (A) Countries where *C. auris* has been reported are presented and categorised by reported association with HCAI outbreaks or evidence of endemicity within a country. **(B)** Countries where *C. auris* has been reported are presented according to the year that the first case was detected. Note: There are many countries where *C. auris* has not yet been reported (white shading); however, this does not mean that it is not present within these countries. Cases of *C. auris* have been detected and reported in Reunion; however, this is not visible on the maps. Maps were prepared in R (v4.3.1) using the package rworldmap. See supplementary appendix 1 for a full list of countries and references used to produce these maps.

References

1. Kim, M.N., et al., *Candida haemulonii and closely related species at 5 university hospitals in Korea: identification, antifungal susceptibility, and clinical features*. Clin Infect Dis, 2009. **48**(6): p. e57-61.
2. Satoh, K., et al., *Candida auris sp. nov., a novel ascomycetous yeast isolated from the external ear canal of an inpatient in a Japanese hospital*. Microbiol Immunol, 2009. **53**(1): p. 41-4.
3. Lee, W.G., et al., *First three reported cases of nosocomial fungemia caused by Candida auris*. J Clin Microbiol, 2011. **49**(9): p. 3139-42.
4. Oh, B.J., et al., *Biofilm formation and genotyping of Candida haemulonii, Candida pseudohaemulonii, and a proposed new species (Candida auris) isolates from Korea*. Med Mycol, 2011. **49**(1): p. 98-102.
5. Shin, J.H., et al., *Detection of amphotericin B resistance in Candida haemulonii and closely related species by use of the Etest, Vitek-2 yeast susceptibility system, and CLSI and EUCAST broth microdilution methods*. J Clin Microbiol, 2012. **50**(6): p. 1852-5.
6. Chowdhary, A., et al., *New clonal strain of Candida auris, Delhi, India*. Emerg Infect Dis, 2013. **19**(10): p. 1670-3.
7. Sarma, S., et al., *Candidemia caused by amphotericin B and fluconazole resistant Candida auris*. Indian J Med Microbiol, 2013. **31**(1): p. 90-1.
8. Chowdhary, A., et al., *Multidrug-resistant endemic clonal strain of Candida auris in India*. Eur J Clin Microbiol Infect Dis, 2014. **33**(6): p. 919-26.
9. Magobo, R.E., et al., *Candida auris-associated candidemia, South Africa*. Emerg Infect Dis, 2014. **20**(7): p. 1250-1.
10. Chakrabarti, A., et al., *Incidence, characteristics and outcome of ICU-acquired candidemia in India*. Intensive Care Med, 2015. **41**(2): p. 285-95.
11. Emara, M., et al., *Candida auris candidemia in Kuwait, 2014*. Emerg Infect Dis, 2015. **21**(6): p. 1091-2.
12. Kumar, D., et al., *Itraconazole-resistant Candida auris with phospholipase, proteinase and hemolysin activity from a case of vulvovaginitis*. J Infect Dev Ctries, 2015. **9**(4): p. 435-7.
13. Calvo, B., et al., *First report of Candida auris in America: Clinical and microbiological aspects of 18 episodes of candidemia*. J Infect, 2016. **73**(4): p. 369-74.
14. Schelenz, S., et al., *First hospital outbreak of the globally emerging Candida auris in a European hospital*. Antimicrob Resist Infect Control, 2016. **5**: p. 35.
15. Sharma, C., et al., *Whole genome sequencing of emerging multidrug resistant Candida auris isolates in India demonstrates low genetic variation*. New Microbes New Infect, 2016. **13**: p. 77-82.
16. Vallabhaneni, S., et al., *Investigation of the First Seven Reported Cases of Candida auris, a Globally Emerging Invasive, Multidrug-Resistant Fungus - United States, May 2013-August 2016*. MMWR Morb Mortal Wkly Rep, 2016. **65**(44): p. 1234-1237.

17. Al-Siyabi, T., et al., *First report of Candida auris in Oman: Clinical and microbiological description of five candidemia cases.* J Infect, 2017. **75**(4): p. 373-376.
18. Azar, M.M., et al., *Donor-Derived Transmission of Candida auris During Lung Transplantation.* Clin Infect Dis, 2017. **65**(6): p. 1040-1042.
19. Ben-Ami, R., et al., *Multidrug-Resistant Candida haemulonii and C. auris, Tel Aviv, Israel.* Emerg Infect Dis, 2017. **23**(1): p. 195-203.
20. Biswal, M., et al., *Controlling a possible outbreak of Candida auris infection: lessons learnt from multiple interventions.* J Hosp Infect, 2017. **97**(4): p. 363-370.
21. Borman, A.M., A. Szekely, and E.M. Johnson, *Isolates of the emerging pathogen Candida auris present in the UK have several geographic origins.* Med Mycol, 2017. **55**(5): p. 563-567.
22. Choi, H.I., et al., *Otomastoiditis caused by Candida auris: Case report and literature review.* Mycoses, 2017. **60**(8): p. 488-492.
23. Lockhart, S.R., et al., *Simultaneous Emergence of Multidrug-Resistant Candida auris on 3 Continents Confirmed by Whole-Genome Sequencing and Epidemiological Analyses.* Clin Infect Dis, 2017. **64**(2): p. 134-140.
24. Mohsin, J., et al., *The first cases of Candida auris candidaemia in Oman.* Mycoses, 2017. **60**(9): p. 569-575.
25. Morales-López, S.E., et al., *Invasive Infections with Multidrug-Resistant Yeast Candida auris, Colombia.* Emerg Infect Dis, 2017. **23**(1): p. 162-164.
26. Rudramurthy, S.M., et al., *Candida auris candidaemia in Indian ICUs: analysis of risk factors.* J Antimicrob Chemother, 2017. **72**(6): p. 1794-1801.
27. Ruiz Gaitán, A.C., et al., *Nosocomial fungemia by Candida auris: First four reported cases in continental Europe.* Rev Iberoam Micol, 2017. **34**(1): p. 23-27.
28. Schwartz, I.S. and G.W. Hammond, *First reported case of multidrug-resistant Candida auris in Canada.* Can Commun Dis Rep, 2017. **43**(7-8): p. 150-153.
29. Tsay, S., et al., *Notes from the Field: Ongoing Transmission of Candida auris in Health Care Facilities - United States, June 2016-May 2017.* MMWR Morb Mortal Wkly Rep, 2017. **66**(19): p. 514-515.
30. Vallabhaneni, S., et al., *Investigation of the First Seven Reported Cases of Candida auris, a Globally Emerging Invasive, Multidrug-Resistant Fungus-United States, May 2013-August 2016.* Am J Transplant, 2017. **17**(1): p. 296-299.
31. Abdalhamid, B., et al., *First report of Candida auris infections from Saudi Arabia.* J Infect Public Health, 2018. **11**(4): p. 598-599.
32. Adams, E., et al., *Candida auris in Healthcare Facilities, New York, USA, 2013-2017.* Emerg Infect Dis, 2018. **24**(10): p. 1816-1824.
33. Alatoom, A., et al., *Persistent candidemia despite appropriate fungal therapy: First case of Candida auris from the United Arab Emirates.* Int J Infect Dis, 2018. **70**: p. 36-37.
34. Araúz, A.B., et al., *Isolation of Candida auris from 9 patients in Central America: Importance of accurate diagnosis and susceptibility testing.* Mycoses, 2018. **61**(1): p. 44-47.
35. Belkin, A., et al., *Candida auris Infection Leading to Nosocomial Transmission, Israel, 2017.* Emerg Infect Dis, 2018. **24**(4): p. 801-804.

36. Bougnoux, M.E., S. Brun, and J.R. Zahar, *Healthcare-associated fungal outbreaks: New and uncommon species, New molecular tools for investigation and prevention.* Antimicrob Resist Infect Control, 2018. **7**: p. 45.
37. Chen, Y., et al., *Emergency of fungemia cases caused by fluconazole-resistant Candida auris in Beijing, China.* J Infect, 2018. **77**(6): p. 561-571.
38. Chow, N.A., et al., *Multiple introductions and subsequent transmission of multidrug-resistant Candida auris in the USA: a molecular epidemiological survey.* Lancet Infect Dis, 2018. **18**(12): p. 1377-1384.
39. Das, S., et al., *Candida auris in critically ill patients: Emerging threat in intensive care unit of hospitals.* J Mycol Med, 2018. **28**(3): p. 514-518.
40. Das, S., et al., *Candida auris colonization in an immunocompetent patient: A new threat in medical ICU.* Med Mycol Case Rep, 2018. **21**: p. 54-56.
41. Escandón, P., et al., *Notes from the Field: Surveillance for Candida auris - Colombia, September 2016-May 2017.* MMWR Morb Mortal Wkly Rep, 2018. **67**(15): p. 459-460.
42. Eyre, D.W., et al., *A Candida auris Outbreak and Its Control in an Intensive Care Setting.* N Engl J Med, 2018. **379**(14): p. 1322-1331.
43. Govender, N.P., et al., *Candida auris in South Africa, 2012-2016.* Emerg Infect Dis, 2018. **24**(11): p. 2036-2040.
44. Iguchi, S., et al., *The Second Candida auris Isolate from Aural Discharge in Japan.* Jpn J Infect Dis, 2018. **71**(2): p. 174-175.
45. Khan, Z., et al., *Increasing prevalence, molecular characterization and antifungal drug susceptibility of serial Candida auris isolates in Kuwait.* PLoS One, 2018. **13**(4): p. e0195743.
46. Khan, Z., et al., *Invasive Candida auris infections in Kuwait hospitals: epidemiology, antifungal treatment and outcome.* Infection, 2018. **46**(5): p. 641-650.
47. Kohlenberg, A., et al., *Candida auris: epidemiological situation, laboratory capacity and preparedness in European Union and European Economic Area countries, 2013 to 2017.* Euro Surveill, 2018. **23**(13).
48. Lesho, E.P., et al., *Importation, Mitigation, and Genomic Epidemiology of Candida auris at a Large Teaching Hospital.* Infect Control Hosp Epidemiol, 2018. **39**(1): p. 53-57.
49. Mathur, P., et al., *Five-year profile of candidaemia at an Indian trauma centre: High rates of Candida auris blood stream infections.* Mycoses, 2018. **61**(9): p. 674-680.
50. Mohd Tap, R., et al., *A Fatal Case of Candida auris and Candida tropicalis Candidemia in Neutropenic Patient.* Mycopathologia, 2018. **183**(3): p. 559-564.
51. Noginskiy, I., et al., *A Case of Multiple Myeloma Presenting as Streptococcus pneumoniae Meningitis with Candida auris Fungemia.* Case Rep Oncol, 2018. **11**(3): p. 705-710.
52. Parra-Giraldo, C.M., et al., *First report of sporadic cases of Candida auris in Colombia.* Int J Infect Dis, 2018. **69**: p. 63-67.
53. Pekard-Amenitsch, S., et al., *Isolation of Candida auris from Ear of Otherwise Healthy Patient, Austria, 2018.* Emerg Infect Dis, 2018. **24**(8): p. 1596-1597.
54. Rhodes, J., et al., *Genomic epidemiology of the UK outbreak of the emerging human fungal pathogen Candida auris.* Emerg Microbes Infect, 2018. **7**(1): p. 43.

55. Riat, A., et al., *First case of Candida auris in Switzerland: discussion about preventive strategies.* Swiss Med Wkly, 2018. **148**: p. w14622.
56. Ruiz-Gaitán, A., et al., *An outbreak due to Candida auris with prolonged colonisation and candidaemia in a tertiary care European hospital.* Mycoses, 2018. **61**(7): p. 498-505.
57. Tan, Y.E. and A.L. Tan, *Arrival of Candida auris Fungus in Singapore: Report of the First 3 Cases.* Ann Acad Med Singap, 2018. **47**(7): p. 260-262.
58. Tian, S., et al., *First cases and risk factors of super yeast Candida auris infection or colonization from Shenyang, China.* Emerg Microbes Infect, 2018. **7**(1): p. 128.
59. Tsay, S., et al., *Approach to the Investigation and Management of Patients With Candida auris, an Emerging Multidrug-Resistant Yeast.* Clin Infect Dis, 2018. **66**(2): p. 306-311.
60. Wang, X., et al., *The first isolate of Candida auris in China: clinical and biological aspects.* Emerg Microbes Infect, 2018. **7**(1): p. 93.
61. Abastabar, M., et al., *Candida auris otomycosis in Iran and review of recent literature.* Mycoses, 2019. **62**(2): p. 101-105.
62. Adam, R.D., et al., *Analysis of Candida auris fungemia at a single facility in Kenya.* Int J Infect Dis, 2019. **85**: p. 182-187.
63. Al Maani, A., et al., *Ongoing Challenges with Healthcare-Associated Candida auris Outbreaks in Oman.* J Fungi (Basel), 2019. **5**(4).
64. Alfouzan, W., et al., *The emerging pathogen Candida auris: A focus on the Middle-Eastern countries.* J Infect Public Health, 2019. **12**(4): p. 451-459.
65. Alobaid, K. and Z. Khan, *Epidemiologic characteristics of adult candidemic patients in a secondary hospital in Kuwait: A retrospective study.* J Mycol Med, 2019. **29**(1): p. 35-38.
66. Armstrong, P.A., et al., *Hospital-Associated Multicenter Outbreak of Emerging Fungus Candida auris, Colombia, 2016.* Emerg Infect Dis, 2019. **25**(7): p. 1339-46.
67. Arun, C.S., et al., *Emergence of Fluconazole-resistant Candida Infections in Diabetic Foot Ulcers: Implications for Public Health.* Indian J Community Med, 2019. **44**(Suppl 1): p. S74-s76.
68. Badri, A.M., Sherfi, S. A., *First Detection of Emergent Fungal Pathogen Candida auris in Khartoum State, Sudan.* Am J Biomed Sci & Res, 2019. **6**(1): p. AJBSR.MS.ID.000982.
69. Barantsevich, N.E., et al., *Emergence of Candida auris in Russia.* J Hosp Infect, 2019. **102**(4): p. 445-448.
70. Biagi, M.J., et al., *Development of High-Level Echinocandin Resistance in a Patient With Recurrent Candida auris Candidemia Secondary to Chronic Candiduria.* Open Forum Infect Dis, 2019. **6**(7): p. ofz262.
71. Brooks, R.B., et al., *Candida auris in a U.S. Patient with Carbapenemase-Producing Organisms and Recent Hospitalization in Kenya.* MMWR Morb Mortal Wkly Rep, 2019. **68**(30): p. 664-666.
72. Castro, L., et al., *Candida auris infection in the central catheter of a patient without sepsis symptoms.* Colomb Med (Cali), 2019. **50**(4): p. 293-298.
73. Ceballos-Garzón, A., et al., *Comparison between MALDI-TOF MS and MicroScan in the identification of emerging and multidrug resistant yeasts in a fourth-level hospital in Bogotá, Colombia.* BMC Microbiol, 2019. **19**(1): p. 106.

74. Chow, N.A., et al., *Potential Fifth Clade of Candida auris, Iran, 2018.* Emerg Infect Dis, 2019. **25**(9): p. 1780-1781.
75. Crea, F., et al., *Isolation of Candida auris from invasive and non-invasive samples of a patient suffering from vascular disease, Italy, July 2019.* Euro Surveill, 2019. **24**(37).
76. Elsawy, A., et al., *The second confirmed case of Candida auris from Saudi Arabia.* J Infect Public Health, 2019. **12**(6): p. 907-908.
77. Escandón, P., et al., *Molecular Epidemiology of Candida auris in Colombia Reveals a Highly Related, Countrywide Colonization With Regional Patterns in Amphotericin B Resistance.* Clin Infect Dis, 2019. **68**(1): p. 15-21.
78. Hamprecht, A., et al., *Candida auris in Germany and Previous Exposure to Foreign Healthcare.* Emerg Infect Dis, 2019. **25**(9): p. 1763-1765.
79. Heath, C.H., et al., *Candida auris Sternal Osteomyelitis in a Man from Kenya Visiting Australia, 2015.* Emerg Infect Dis, 2019. **25**(1): p. 192-194.
80. Khatamzas, E., H. Madder, and K. Jeffery, *Neurosurgical device-associated infections due to Candida auris - Three cases from a single tertiary center.* J Infect, 2019. **78**(5): p. 409-421.
81. Kumar, J., et al., *Environmental Contamination with Candida Species in Multiple Hospitals Including a Tertiary Care Hospital with a Candida auris Outbreak.* Pathog Immun, 2019. **4**(2): p. 260-270.
82. Kwon, Y.J., et al., *Candida auris Clinical Isolates from South Korea: Identification, Antifungal Susceptibility, and Genotyping.* J Clin Microbiol, 2019. **57**(4).
83. Long, S.W., et al., *Draft Genome Sequence of Candida auris Strain LOM, a Human Clinical Isolate from Greater Metropolitan Houston, Texas.* Microbiol Resour Announc, 2019. **8**(25).
84. Moreno, M.V., et al., *[First isolation de Candida auris in Chile].* Rev Chilena Infectol, 2019. **36**(6): p. 767-773.
85. O'Connor, C., et al., *Candida auris outbreak on a vascular ward - the unexpected arrival of an anticipated pathogen.* J Hosp Infect, 2019. **103**(1): p. 106-108.
86. Ong, C.W., et al., *Diagnosis, management and prevention of Candida auris in hospitals: position statement of the Australasian Society for Infectious Diseases.* Intern Med J, 2019. **49**(10): p. 1229-1243.
87. Park, J.Y., et al., *Management of Patients with Candida auris Fungemia at Community Hospital, Brooklyn, New York, USA, 2016-2018(1).* Emerg Infect Dis, 2019. **25**(3): p. 601-602.
88. Pfaller, M.A., et al., *Twenty Years of the SENTRY Antifungal Surveillance Program: Results for Candida Species From 1997-2016.* Open Forum Infect Dis, 2019. **6**(Suppl 1): p. S79-s94.
89. Rhodes, J. and M.C. Fisher, *Global epidemiology of emerging Candida auris.* Curr Opin Microbiol, 2019. **52**: p. 84-89.
90. Roberts, S.C., et al., *Successful treatment of a Candida auris intra-articular infection.* Emerg Microbes Infect, 2019. **8**(1): p. 866-868.
91. Ruiz-Gaitán, A., et al., *Detection and treatment of Candida auris in an outbreak situation: risk factors for developing colonization and candidemia by this new species in critically ill patients.* Expert Rev Anti Infect Ther, 2019. **17**(4): p. 295-305.

92. Ruiz-Gaitán, A.C., et al., *Outbreak of Candida auris in Spain: A comparison of antifungal activity by three methods with published data.* Int J Antimicrob Agents, 2019. **53**(5): p. 541-546.
93. Sana, F., et al., *Candida auris outbreak report from Pakistan: a success story of infection control in ICUs of a tertiary care hospital.* J Hosp Infect, 2019. **103**(1): p. 108-110.
94. Sayeed, M.A., et al., *Clinical spectrum and factors impacting outcome of Candida auris: a single center study from Pakistan.* BMC Infect Dis, 2019. **19**(1): p. 384.
95. Shenoy, V., et al., *Panophthalmitis From Candida auris.* Ann Intern Med, 2019. **171**(12): p. 941-943.
96. Stathi, A., et al., *Isolation of Candida auris from cystic fibrosis patient, Greece, April 2019.* Euro Surveill, 2019. **24**(29).
97. Tan, Y.E., et al., *Candida auris in Singapore: Genomic epidemiology, antifungal drug resistance, and identification using the updated 8.01 VITEK(Ⓡ)2 system.* Int J Antimicrob Agents, 2019. **54**(6): p. 709-715.
98. Tang, H.J., et al., *Emergence of multidrug-resistant Candida auris in Taiwan.* Int J Antimicrob Agents, 2019. **53**(5): p. 705-706.
99. Taori, S.K., et al., *Candida auris outbreak: Mortality, interventions and cost of sustaining control.* J Infect, 2019. **79**(6): p. 601-611.
100. van Schalkwyk, E., et al., *Epidemiologic Shift in Candidemia Driven by Candida auris, South Africa, 2016-2017(1).* Emerg Infect Dis, 2019. **25**(9): p. 1698-1707.
101. Vogelzang, E.H., et al., *The First Two Cases of Candida auris in The Netherlands.* J Fungi (Basel), 2019. **5**(4).
102. Woodworth, M.H., et al., *Sentinel Case of Candida auris in the Western United States Following Prolonged Occult Colonization in a Returned Traveler from India.* Microb Drug Resist, 2019. **25**(5): p. 677-680.
103. Ahmad, S., et al., *Candida auris in various hospitals across Kuwait and their susceptibility and molecular basis of resistance to antifungal drugs.* Mycoses, 2020. **63**(1): p. 104-112.
104. Alfouzan, W., et al., *Molecular Epidemiology of Candida Auris Outbreak in a Major Secondary-Care Hospital in Kuwait.* J Fungi (Basel), 2020. **6**(4).
105. AlJindan, R., et al., *Drug Resistance-Associated Mutations in ERG11 of Multidrug-Resistant Candida auris in a Tertiary Care Hospital of Eastern Saudi Arabia.* J Fungi (Basel), 2020. **7**(1).
106. Almaghrabi, R.S., et al., *Molecular characterisation and clinical outcomes of Candida auris infection: Single-centre experience in Saudi Arabia.* Mycoses, 2020. **63**(5): p. 452-460.
107. Álvarez Duarte, E. and V. Salas, *In vitro activity of isavuconazole against clinically isolated yeasts from Chile.* Braz J Microbiol, 2020. **51**(4): p. 1801-1805.
108. Anwar, S., et al., *Candida auris-an impending threat: A case report from home.* Am J Infect Control, 2020. **48**(11): p. 1407-1408.
109. Arensman, K., et al., *Clinical Outcomes of Patients Treated for Candida auris Infections in a Multisite Health System, Illinois, USA.* Emerg Infect Dis, 2020. **26**(5): p. 876-880.

110. Bajpai, V., et al., *Multidrug-Resistant Candida auris Fungemia in Critical Care Units: Experience from a Tertiary Care Hospital in India.* Microb Drug Resist, 2020. **26**(2): p. 145-149.
111. Barantsevich, N.E., et al., *Candida auris Bloodstream Infections in Russia.* Antibiotics (Basel), 2020. **9**(9).
112. Biswas, C., et al., *Genetic Heterogeneity of Australian Candida auris Isolates: Insights From a Nonoutbreak Setting Using Whole-Genome Sequencing.* Open Forum Infect Dis, 2020. **7**(5): p. ofaa158.
113. Breazzano, M.P., et al., *Candida auris and endogenous panophthalmitis: clinical and histopathological features.* Am J Ophthalmol Case Rep, 2020. **19**: p. 100738.
114. Caceres, D.H., et al., *Case-Case Comparison of Candida auris Versus Other Candida Species Bloodstream Infections: Results of an Outbreak Investigation in Colombia.* Mycopathologia, 2020. **185**(5): p. 917-923.
115. Chakrabarti, A., et al., *Characteristics, outcome and risk factors for mortality of paediatric patients with ICU-acquired candidemia in India: A multicentre prospective study.* Mycoses, 2020. **63**(11): p. 1149-1163.
116. Chandramati, J., et al., *Neonatal Candida auris infection: Management and prevention strategies - A single centre experience.* J Paediatr Child Health, 2020. **56**(10): p. 1565-1569.
117. Chowdhary, A., et al., *Multidrug-Resistant Candida auris Infections in Critically Ill Coronavirus Disease Patients, India, April-July 2020.* Emerg Infect Dis, 2020. **26**(11): p. 2694-2696.
118. Dewaele, K., et al., *First case of Candida auris infection in Belgium in a surgical patient from Kuwait.* Acta Clin Belg, 2020. **75**(3): p. 221-228.
119. Dutta, S., et al., *Detection of Candida auris and its antifungal susceptibility: first report from Bangladesh.* IMC Journal of Medical Science, 2020. **13**(2): p. 18-22.
120. Farooqi, J.Q., et al., *Outbreak investigation of Candida auris at a tertiary care hospital in Karachi, Pakistan.* J Infect Prev, 2020. **21**(5): p. 189-195.
121. García, C.S., et al., *Candida auris: report of an outbreak.* Enferm Infecc Microbiol Clin (Engl Ed), 2020. **38 Suppl 1**: p. 39-44.
122. Garcia Rivera, M.V., J.J. Heyl, and M.C. Oh, *Candida Auris Urinary Tract Infection in a Nursing Home Patient With Multicomorbidities.* Cureus, 2020. **12**(12): p. e12322.
123. Garcia-Bustos, V., et al., *A clinical predictive model of candidaemia by Candida auris in previously colonized critically ill patients.* Clin Microbiol Infect, 2020. **26**(11): p. 1507-1513.
124. Garcia-Jeldes, H.F., et al., *Prevalence of Candida auris in Canadian acute care hospitals among at-risk patients, 2018.* Antimicrob Resist Infect Control, 2020. **9**(1): p. 82.
125. Jung, J., et al., *Candida auris colonization or infection of the ear: A single-center study in South Korea from 2016 to 2018.* Med Mycol, 2020. **58**(1): p. 124-127.
126. Kaur, H., et al., *Candidaemia in a tertiary care centre of developing country: Monitoring possible change in spectrum of agents and antifungal susceptibility.* Indian J Med Microbiol, 2020. **38**(1): p. 110-116.
127. Khan, A., et al., *Simultaneous Infection with Enterobacteriaceae and Pseudomonas aeruginosa Harboring Multiple Carbapenemases in a Returning*

Traveler Colonized with Candida auris. Antimicrob Agents Chemother, 2020. **64**(2).
128. Lane, C.R., et al., *Incursions of Candida auris into Australia, 2018.* Emerg Infect Dis, 2020. **26**(6): p. 1326-1328.
129. Levy, Y., et al., *Case Report: Emergence of Candida auris in the Indian Ocean Region.* Am J Trop Med Hyg, 2020. **104**(2): p. 739-743.
130. Lingas, E., et al., *A Case of Candida auris Candidemia in an Immunocompetent Traumatic Brain Injury Patient Post Ventriculoperitoneal Shunt and Peripherally Inserted Central Catheter Line.* Cureus, 2020. **12**(6): p. e8850.
131. Long, S.W., et al., *Human Infections Caused by Clonally Related African Clade (Clade III) Strains of Candida auris in the Greater Houston Region.* J Clin Microbiol, 2020. **58**(7).
132. Magobo, R., et al., *Multilocus sequence typing of azole-resistant Candida auris strains, South Africa.* S Afr J Infect Dis, 2020. **35**(1): p. 116.
133. Meena, S., et al., *Candida auris emergence in the Himalayan foothills: First case report from Uttarakhand, India.* Curr Med Mycol, 2020. **6**(1): p. 47-50.
134. Mohsin, J., et al., *A Cluster of Candida auris Blood Stream Infections in a Tertiary Care Hospital in Oman from 2016 to 2019.* Antibiotics (Basel), 2020. **9**(10).
135. Mulet Bayona, J.V., et al., *Characteristics and Management of Candidaemia Episodes in an Established Candida auris Outbreak.* Antibiotics (Basel), 2020. **9**(9).
136. Ninan, M.M., et al., *Candida auris: Clinical profile, diagnostic challenge and susceptibility pattern: Experience from a tertiary-care centre in South India.* J Glob Antimicrob Resist, 2020. **21**: p. 181-185.
137. O'Brien, B., S. Chaturvedi, and V. Chaturvedi, *In Vitro Evaluation of Antifungal Drug Combinations against Multidrug-Resistant Candida auris Isolates from New York Outbreak.* Antimicrob Agents Chemother, 2020. **64**(4).
138. O'Brien, B., et al., *Pan-resistant Candida auris: New York subcluster susceptible to antifungal combinations.* Lancet Microbe, 2020. **1**(5): p. e193-e194.
139. Ostrowsky, B., et al., *Candida auris Isolates Resistant to Three Classes of Antifungal Medications - New York, 2019.* MMWR Morb Mortal Wkly Rep, 2020. **69**(1): p. 6-9.
140. Pacilli, M., et al., *Regional Emergence of Candida auris in Chicago and Lessons Learned From Intensive Follow-up at 1 Ventilator-Capable Skilled Nursing Facility.* Clin Infect Dis, 2020. **71**(11): p. e718-e725.
141. Pchelin, I.M., et al., *Whole genome sequence of first Candida auris strain, isolated in Russia.* Med Mycol, 2020. **58**(3): p. 414-416.
142. Plachouras, D., et al., *Candida auris: epidemiological situation, laboratory capacity and preparedness in the European Union and European Economic Area*, January 2018 to May 2019.* Euro Surveill, 2020. **25**(12).
143. Sayeed, M.A., et al., *Comparison of risk factors and outcomes of Candida auris candidemia with non-Candida auris candidemia: A retrospective study from Pakistan.* Med Mycol, 2020. **58**(6): p. 721-729.
144. Shastri, P.S., et al., *Candida auris candidaemia in an intensive care unit - Prospective observational study to evaluate epidemiology, risk factors, and outcome.* J Crit Care, 2020. **57**: p. 42-48.

145. Supreeth, S., et al., *First Report of Candida auris Spondylodiscitis in Oman: A Rare Presentation*. World Neurosurg, 2020. **135**: p. 335-338.
146. Theodoropoulos, N.M., et al., *Candida auris outbreak involving liver transplant recipients in a surgical intensive care unit*. Am J Transplant, 2020. **20**(12): p. 3673-3679.
147. Vuichard-Gysin, D., et al., *Candida auris - recommendations on infection prevention and control measures in Switzerland*. Swiss Med Wkly, 2020. **150**: p. w20297.
148. Worth, L.J., et al., *Candida auris in an Australian health care facility: importance of screening high risk patients*. Med J Aust, 2020. **212**(11): p. 510-511.e1.
149. Zhu, Y., et al., *In Vitro Activity of Manogepix against Multidrug-Resistant and Panresistant Candida auris from the New York Outbreak*. Antimicrob Agents Chemother, 2020. **64**(11).
150. Zhu, Y., et al., *Laboratory Analysis of an Outbreak of Candida auris in New York from 2016 to 2018: Impact and Lessons Learned*. J Clin Microbiol, 2020. **58**(4).
151. Zuo, T., et al., *Alterations in Fecal Fungal Microbiome of Patients With COVID-19 During Time of Hospitalization until Discharge*. Gastroenterology, 2020. **159**(4): p. 1302-1310.e5.
152. Al-Jindan, R. and D.M. Al-Eraky, *Two Cases of the Emerging Candida auris in a university hospital from Saudi Arabia*. Saudi J Med Med Sci, 2021. **9**(1): p. 71-74.
153. Al-Rashdi, A., et al., *Characteristics, Risk Factors, and Survival Analysis of Candida auris Cases: Results of One-Year National Surveillance Data from Oman*. J Fungi (Basel), 2021. **7**(1).
154. Alashqar, M.B., et al., *A Case Report of a Candida auris Infection in Saudi Arabia*. Cureus, 2021. **13**(5): p. e15240.
155. Allaw, F., et al., *First Candida auris Outbreak during a COVID-19 Pandemic in a Tertiary-Care Center in Lebanon*. Pathogens, 2021. **10**(2).
156. Alobaid, K., et al., *Epidemiology of Candidemia in Kuwait: A Nationwide, Population-Based Study*. J Fungi (Basel), 2021. **7**(8).
157. Alshamrani, M.M., et al., *Management of Candida auris outbreak in a tertiary-care setting in Saudi Arabia*. Infect Control Hosp Epidemiol, 2021. **42**(2): p. 149-155.
158. Alvarado-Socarras, J.L., et al., *A Cluster of Neonatal Infections Caused by Candida auris at a Large Referral Center in Colombia*. J Pediatric Infect Dis Soc, 2021. **10**(5): p. 549-555.
159. Ayala-Gaytán, J.J., et al., *First case of Candida auris isolated from the bloodstream of a Mexican patient with serious gastrointestinal complications from severe endometriosis*. Infection, 2021. **49**(3): p. 523-525.
160. Bacchani, D., et al., *Prevalence, epidemiology and clinical outcome of Candida auris infections: Experience from a tertiary care hospital in Jaipur*. Trop Doct, 2021. **51**(4): p. 508-513.
161. Berrio, I., et al., *Bloodstream Infections With Candida auris Among Children in Colombia: Clinical Characteristics and Outcomes of 34 Cases*. J Pediatric Infect Dis Soc, 2021. **10**(2): p. 151-154.
162. Bölükbaşı, Y., et al., *[First Case of COVID-19 Positive Candida auris Fungemia in Turkey]*. Mikrobiyol Bul, 2021. **55**(4): p. 648-655.

163. Borgio, J.F., et al., *Emerging Status of Multidrug-Resistant Bacteria and Fungi in the Arabian Peninsula.* Biology (Basel), 2021. **10**(11).
164. de Almeida, J.N., Jr., et al., *Emergence of Candida auris in Brazil in a COVID-19 Intensive Care Unit.* J Fungi (Basel), 2021. **7**(3).
165. de Jong, A.W., et al., *Nanopore Genome Sequencing and Variant Analysis of the Susceptible Candida auris Strain L1537/2020, Salvador, Brazil.* Mycopathologia, 2021. **186**(6): p. 883-887.
166. Desnos-Ollivier, M., A. Fekkar, and S. Bretagne, *Earliest case of Candida auris infection imported in 2007 in Europe from India prior to the 2009 description in Japan.* J Mycol Med, 2021. **31**(3): p. 101139.
167. Di Pilato, V., et al., *Molecular Epidemiological Investigation of a Nosocomial Cluster of C. auris: Evidence of Recent Emergence in Italy and Ease of Transmission during the COVID-19 Pandemic.* J Fungi (Basel), 2021. **7**(2).
168. Eckbo, E.J., et al., *First reported outbreak of the emerging pathogen Candida auris in Canada.* Am J Infect Control, 2021. **49**(6): p. 804-807.
169. Fan, S., et al., *A biological and genomic comparison of a drug-resistant and a drug-susceptible strain of Candida auris isolated from Beijing, China.* Virulence, 2021. **12**(1): p. 1388-1399.
170. Farooqi, J., et al., *Comparison of β-D-Glucan levels between Candida auris and other Candida species at the time of candidaemia: a retrospective study.* Clin Microbiol Infect, 2021. **27**(10): p. 1519.e1-1519.e5.
171. Ferrer Gómez, C., et al., *Analysis of Candida auris candidemia cases in an Intensive Care Unit of a tertiary hospital.* Rev Esp Anestesiol Reanim (Engl Ed), 2021. **68**(8): p. 431-436.
172. Goravey, W., et al., *Ominous combination: COVID-19 disease and Candida auris fungemia-Case report and review of the literature.* Clin Case Rep, 2021. **9**(9): p. e04827.
173. Hanson, B.M., et al., *Candida auris Invasive Infections during a COVID-19 Case Surge.* Antimicrob Agents Chemother, 2021. **65**(10): p. e0114621.
174. Huang, X., et al., *Skin Metagenomic Sequence Analysis of Early Candida auris Outbreaks in U.S. Nursing Homes.* mSphere, 2021. **6**(4): p. e0028721.
175. Karmarkar, E.N., et al., *Rapid Assessment and Containment of Candida auris Transmission in Postacute Care Settings-Orange County, California, 2019.* Ann Intern Med, 2021. **174**(11): p. 1554-1562.
176. Kömeç, S., et al., *[Three Candida auris Case Reports from Istanbul, Turkey].* Mikrobiyol Bul, 2021. **55**(3): p. 452-460.
177. Kurt, A.F., et al., *Candida auris Fungemia and a local spread taken under control with infection control measures: First report from Turkey.* Indian J Med Microbiol, 2021. **39**(2): p. 228-230.
178. Lyman, M., et al., *Notes from the Field: Transmission of Pan-Resistant and Echinocandin-Resistant Candida auris in Health Care Facilities - Texas and the District of Columbia, January-April 2021.* MMWR Morb Mortal Wkly Rep, 2021. **70**(29): p. 1022-1023.
179. Meawed, T.E., et al., *Bacterial and fungal ventilator associated pneumonia in critically ill COVID-19 patients during the second wave.* J Infect Public Health, 2021. **14**(10): p. 1375-1380.

180. Mesini, A., et al., *First Case of Candida auris Colonization in a Preterm, Extremely Low-Birth-Weight Newborn after Vaginal Delivery.* J Fungi (Basel), 2021. **7**(8).
181. Moin, S., et al., *C. auris and non-C. auris candidemia in hospitalized adult and pediatric COVID-19 patients; single center data from Pakistan.* Med Mycol, 2021. **59**(12): p. 1238-1242.
182. Mulet Bayona, J.V., et al., *Impact of the SARS-CoV-2 Pandemic in Candidaemia, Invasive Aspergillosis and Antifungal Consumption in a Tertiary Hospital.* J Fungi (Basel), 2021. **7**(6).
183. Mulet-Bayona, J.V., et al., *Recurrent candidemia and isolation of echinocandin-resistant Candida auris in a patient with a long-term central catheter.* Enferm Infecc Microbiol Clin (Engl Ed), 2021.
184. Naicker, S.D., et al., *Clade distribution of Candida auris in South Africa using whole genome sequencing of clinical and environmental isolates.* Emerg Microbes Infect, 2021. **10**(1): p. 1300-1308.
185. Nobrega de Almeida, J., Jr., et al., *Axillary Digital Thermometers uplifted a multidrug-susceptible Candida auris outbreak among COVID-19 patients in Brazil.* Mycoses, 2021. **64**(9): p. 1062-1072.
186. Pandya, N., et al., *International Multicentre Study of Candida auris Infections.* J Fungi (Basel), 2021. **7**(10).
187. Prestel, C., et al., *Candida auris Outbreak in a COVID-19 Specialty Care Unit - Florida, July-August 2020.* MMWR Morb Mortal Wkly Rep, 2021. **70**(2): p. 56-57.
188. Price, T.K., et al., *Genomic Characterizations of Clade III Lineage of Candida auris, California, USA.* Emerg Infect Dis, 2021. **27**(4): p. 1223-1227.
189. Proctor, D.M., et al., *Integrated genomic, epidemiologic investigation of Candida auris skin colonization in a skilled nursing facility.* Nat Med, 2021. **27**(8): p. 1401-1409.
190. Rajni, E., et al., *A High Frequency of Candida auris Blood Stream Infections in Coronavirus Disease 2019 Patients Admitted to Intensive Care Units, Northwestern India: A Case Control Study.* Open Forum Infect Dis, 2021. **8**(12): p. ofab452.
191. Rakiro, J., et al., *Microbial coinfections and superinfections in critical COVID-19: a Kenyan retrospective cohort analysis.* IJID Reg, 2021. **1**: p. 41-46.
192. Reimer-McAtee, M., et al., *Successful implementation of the CDC recommendations during the care of 2 patients with Candida auris in in-patient rehabilitation and intensive care settings.* Am J Infect Control, 2021. **49**(4): p. 525-527.
193. Reslan, L., et al., *Molecular Characterization of Candida auris Isolates at a Major Tertiary Care Center in Lebanon.* Front Microbiol, 2021. **12**: p. 770635.
194. Rossow, J., et al., *Factors Associated With Candida auris Colonization and Transmission in Skilled Nursing Facilities With Ventilator Units, New York, 2016-2018.* Clin Infect Dis, 2021. **72**(11): p. e753-e760.
195. Ruiz-Azcona, L., et al., *Isolation of Candida auris in large hospitals in the Autonomous Community of Valencia; population-based study (2013-2017).* Rev Iberoam Micol, 2021. **38**(3): p. 141-144.

196. Salah, H., et al., *Genomic Epidemiology of Candida auris in Qatar Reveals Hospital Transmission Dynamics and a South Asian Origin.* J Fungi (Basel), 2021. **7**(3).
197. Sathyapalan, D.T., et al., *Evaluating the measures taken to contain a Candida auris outbreak in a tertiary care hospital in South India: an outbreak investigational study.* BMC Infect Dis, 2021. **21**(1): p. 425.
198. Senok, A., et al., *Coinfections in Patients Hospitalized with COVID-19: A Descriptive Study from the United Arab Emirates.* Infect Drug Resist, 2021. **14**: p. 2289-2296.
199. Shaukat, A., et al., *Experience of treating Candida auris cases at a general hospital in the state of Qatar.* IDCases, 2021. **23**: p. e01007.
200. Shuping, L., et al., *Epidemiology of Culture-confirmed Candidemia Among Hospitalized Children in South Africa, 2012-2017.* Pediatr Infect Dis J, 2021. **40**(8): p. 730-737.
201. Sridharan, S., et al., *Clinical Profile of Non-neutropenic Patients with Invasive Candidiasis: A Retrospective Study in a Tertiary Care Center.* Indian J Crit Care Med, 2021. **25**(3): p. 267-272.
202. Steinmann, J., et al., *Two Candida auris Cases in Germany with No Recent Contact to Foreign Healthcare-Epidemiological and Microbiological Investigations.* J Fungi (Basel), 2021. **7**(5).
203. Taghizadeh Armaki, M., et al., *First fluconazole-resistant Candida auris isolated from fungal otitis in Iran.* Curr Med Mycol, 2021. **7**(1): p. 51-54.
204. Tian, S., et al., *Genomic epidemiology of Candida auris in a general hospital in Shenyang, China: a three-year surveillance study.* Emerg Microbes Infect, 2021. **10**(1): p. 1088-1096.
205. Tse, H., et al., *Draft Genome Sequences of 19 Clinical Isolates of Candida auris from Hong Kong.* Microbiol Resour Announc, 2021. **10**(1).
206. Umamaheshwari, S., et al., *Clinical profile, antifungal susceptibility, and molecular characterization of Candida auris isolated from patients in a South Indian surgical ICU.* J Mycol Med, 2021. **31**(4): p. 101176.
207. Villanueva-Lozano, H., et al., *Outbreak of Candida auris infection in a COVID-19 hospital in Mexico.* Clin Microbiol Infect, 2021. **27**(5): p. 813-6.
208. Yadav, A., et al., *Colonisation and Transmission Dynamics of Candida auris among Chronic Respiratory Diseases Patients Hospitalised in a Chest Hospital, Delhi, India: A Comparative Analysis of Whole Genome Sequencing and Microsatellite Typing.* J Fungi (Basel), 2021. **7**(2).
209. Al-Obaid, I., et al., *Fatal Breakthrough Candidemia in an Immunocompromised Patient in Kuwait Due to Candida auris Exhibiting Reduced Susceptibility to Echinocandins and Carrying a Novel Mutation in Hotspot-1 of FKS1.* J Fungi (Basel), 2022. **8**(3).
210. Alanio, A., et al., *First Patient-to-Patient Intrahospital Transmission of Clade I Candida auris in France Revealed after a Two-Month Incubation Period.* Microbiol Spectr, 2022. **10**(5): p. e0183322.
211. Allaw, F., et al., *COVID-19 and C. auris: A Case-Control Study from a Tertiary Care Center in Lebanon.* Microorganisms, 2022. **10**(5).
212. Asadzadeh, M., et al., *Molecular characterisation of Candida auris isolates from immunocompromised patients in a tertiary-care hospital in Kuwait reveals a*

novel mutation in FKS1 conferring reduced susceptibility to echinocandins. Mycoses, 2022. **65**(3): p. 331-343.
213. Austin, L., et al., *Novel case of Candida auris in the Veterans Health Administration and in the state of South Carolina.* Am J Infect Control, 2022. **50**(11): p. 1258-1262.
214. Bagheri Lankarani, K., et al., *Candida auris: outbreak fungal pathogen in COVID-19 pandemic: a systematic review and meta-analysis.* Iran J Microbiol, 2022. **14**(3): p. 276-284.
215. Bilal, H., et al., *Distribution and antifungal susceptibility pattern of Candida species from mainland China: A systematic analysis.* Virulence, 2022. **13**(1): p. 1573-1589.
216. Bing, J., et al., *A case of Candida auris candidemia in Xiamen, China, and a comparative analysis of clinical isolates in China.* Mycology, 2022. **13**(1): p. 68-75.
217. Briano, F., et al., *Candida auris Candidemia in Critically Ill, Colonized Patients: Cumulative Incidence and Risk Factors.* Infect Dis Ther, 2022. **11**(3): p. 1149-1160.
218. Chibabhai, V., *Incidence of candidemia and prevalence of azole-resistant candidemia at a tertiary South African hospital - A retrospective laboratory analysis 2016-2020.* S Afr J Infect Dis, 2022. **37**(1): p. 326.
219. Corcione, S., et al., *First Cases of Candida auris in a Referral Intensive Care Unit in Piedmont Region, Italy.* Microorganisms, 2022. **10**(8).
220. Daneshnia, F., et al., *Determinants of fluconazole resistance and echinocandin tolerance in C. parapsilosis isolates causing a large clonal candidemia outbreak among COVID-19 patients in a Brazilian ICU.* Emerg Microbes Infect, 2022. **11**(1): p. 2264-2274.
221. De Luca, D.G., et al., *Four genomic clades of Candida auris identified in Canada, 2012-2019.* Med Mycol, 2022. **60**(1).
222. Deshkar, S., et al., *Identification and Antifungal Drug Susceptibility Pattern of Candida auris in India.* J Glob Infect Dis, 2022. **14**(4): p. 131-135.
223. Du, H., et al., *Candida auris infections in China.* Virulence, 2022. **13**(1): p. 589-591.
224. Escandón, P., et al., *Laboratory-based surveillance of Candida auris in Colombia, 2016-2020.* Mycoses, 2022. **65**(2): p. 222-225.
225. Fathima, K., et al., *Epidemiological Analysis And Successful Management Of A Candida Auris Outbreak In A Secondary Care Hospital Setting In Saudi Arabia; An Outbreak Report.* J Ayub Med Coll Abbottabad, 2022. **34(Suppl 1)**(3): p. S711-s713.
226. Gautam, S., et al., *Case Report: Secondary Hemophagocytic Lymphohistiocytosis (sHLH) and Candida auris Fungemia in Post-acute COVID-19 Syndrome: A Clinical Challenge.* Front Med (Lausanne), 2022. **9**: p. 835421.
227. González-Durán, E., et al., *The use of readily available laboratory tests for the identification of the emerging yeast Candida auris in Mexico.* Arch Microbiol, 2022. **204**(9): p. 592.
228. Hinrichs, C., et al., *Successful control of Candida auris transmission in a German COVID-19 intensive care unit.* Mycoses, 2022. **65**(6): p. 643-649.

229. Imtiaz, F., M. Tariq, and S. Noor Ul Hasan, *Candida auris case in Karachi, a public health threat ahead.* J Pak Med Assoc, 2022. **72**(6): p. 1248.
230. Jacobs, S.E., et al., *Candida auris Pan-Drug-Resistant to Four Classes of Antifungal Agents.* Antimicrob Agents Chemother, 2022. **66**(7): p. e0005322.
231. Kilburn, S., et al., *Antifungal Resistance Trends of Candida auris Clinical Isolates in New York and New Jersey from 2016 to 2020.* Antimicrob Agents Chemother, 2022. **66**(3): p. e0224221.
232. Kohlenberg, A., D.L. Monnet, and D. Plachouras, *Increasing number of cases and outbreaks caused by Candida auris in the EU/EEA, 2020 to 2021.* Euro Surveill, 2022. **27**(46).
233. Mikulska, M., et al., *Sensitivity of Serum Beta-D-Glucan in Candidemia According to Candida Species Epidemiology in Critically Ill Patients Admitted to the Intensive Care Unit.* J Fungi (Basel), 2022. **8**(9).
234. Mirhendi, H., et al., *South Asian (Clade I) Candida auris meningitis in a paediatric patient in Iran with a review of the literature.* Mycoses, 2022. **65**(2): p. 134-139.
235. Mulet-Bayona, J.V., et al., *Recurrent candidemia and isolation of echinocandin-resistant Candida auris in a patient with a long-term central catheter.* Enferm Infecc Microbiol Clin (Engl Ed), 2022. **40**(6): p. 334-335.
236. Okoye, C.A., E. Nweze, and C. Ibe, *Invasive candidiasis in Africa, what is the current picture?* Pathog Dis, 2022. **80**(1).
237. Oladele, R., et al., *Emergence and Genomic Characterization of Multidrug Resistant Candida auris in Nigeria, West Africa.* J Fungi (Basel), 2022. **8**(8).
238. Parak, A., S.L. Stacey, and V. Chibabhai, *Clinical and laboratory features of patients with Candida auris cultures, compared to other Candida, at a South African Hospital.* J Infect Dev Ctries, 2022. **16**(1): p. 213-221.
239. Piatti, G., et al., *Colonization by Candida auris in critically ill patients: role of cutaneous and rectal localization during an outbreak.* J Hosp Infect, 2022. **120**: p. 85-89.
240. Prayag, P.S., et al., *The Dominance of Candida auris: A Single-center Experience of 79 Episodes of Candidemia from Western India.* Indian J Crit Care Med, 2022. **26**(5): p. 560-563.
241. Rajni, E., et al., *A complete clinico-epidemiological and microbiological profile of candidemia cases in a tertiary-care hospital in Western India.* Antimicrob Steward Healthc Epidemiol, 2022. **2**(1): p. e37.
242. Rajni, E., et al., *Risk Factors for Candidemia in Intensive Care Unit: A Matched Case Control Study from North-Western India.* Acta Medica (Hradec Kralove), 2022. **65**(3): p. 83-88.
243. Reque, J., et al., *Candida auris Invasive Infection after Kidney Transplantation.* Case Rep Nephrol, 2022. **2022**: p. 6007607.
244. Riera, F.O., et al., *Invasive Candidiasis: Update and Current Challenges in the Management of This Mycosis in South America.* Antibiotics (Basel), 2022. **11**(7).
245. Rkieh, L., et al., *Outcomes of caspofungin use in the treatment of Candida-related urinary tract infections, a case series.* IDCases, 2022. **28**: p. e01510.
246. Rybak, J.M., et al., *In vivo emergence of high-level resistance during treatment reveals the first identified mechanism of amphotericin B resistance in Candida auris.* Clin Microbiol Infect, 2022. **28**(6): p. 838-843.

247. Safari, F., et al., *A Chronic Autochthonous Fifth Clade Case of Candida auris Otomycosis in Iran*. Mycopathologia, 2022. **187**(1): p. 121-127.
248. Safari, F., et al., *Molecular investigation of the incidence of Candida auris infections at selected hospitals in Iran*. Mycoses, 2022. **65**(12): p. 1137-1145.
249. Saha, D., et al., *The Spectrum of Pathogenic Yeast Infection in a Tertiary Care Hospital in Assam, India*. Cureus, 2022. **14**(11): p. e31512.
250. Sathi, F.A., et al., *Prevalence and Antifungal Susceptibility of Clinically Relevant Candida Species, Identification of Candida auris and Kodamaea ohmeri in Bangladesh*. Trop Med Infect Dis, 2022. **7**(9).
251. Southwick, K., et al., *A description of the first Candida auris-colonized individuals in New York State, 2016-2017*. Am J Infect Control, 2022. **50**(3): p. 358-360.
252. Spruijtenburg, B., et al., *Confirmation of fifth Candida auris clade by whole genome sequencing*. Emerg Microbes Infect, 2022. **11**(1): p. 2405-2411.
253. Taori, S.K., et al., *First experience of implementing Candida auris real-time PCR for surveillance in the UK: detection of multiple introductions with two international clades and improved patient outcomes*. J Hosp Infect, 2022. **127**: p. 111-120.
254. Theut, M., et al., *[The first two cases of Candida auris in Denmark]*. Ugeskr Laeger, 2022. **184**(16).
255. Thoma, R., et al., *The challenge of preventing and containing outbreaks of multidrug-resistant organisms and Candida auris during the coronavirus disease 2019 pandemic: report of a carbapenem-resistant Acinetobacter baumannii outbreak and a systematic review of the literature*. Antimicrob Resist Infect Control, 2022. **11**(1): p. 12.
256. Tsai, Y.T., et al., *The first invasive Candida auris infection in Taiwan*. Emerg Microbes Infect, 2022. **11**(1): p. 1867-1875.
257. Vaseghi, N., et al., *Global prevalence and subgroup analyses of coronavirus disease (COVID-19) associated Candida auris infections (CACa): A systematic review and meta-analysis*. Mycoses, 2022. **65**(7): p. 683-703.
258. Vu, C.A., et al., *Challenges and opportunities in stewardship among solid organ transplant recipients with Candida auris bloodstream infections*. Transpl Infect Dis, 2022. **24**(5): p. e13919.
259. Zerrouki, H., et al., *Emergence of Candida auris in intensive care units in Algeria*. Mycoses, 2022. **65**(7): p. 753-759.
260. Ahmadi, B., et al., *An Autochthonous Susceptible Candida auris Clade I Otomycosis Case in Iran*. J Fungi (Basel), 2023. **9**(11).
261. Al-Ramahi, J.W., et al. *Report of the First Case of Candida auris Identified in Jordan*. Microbiology Research, 2023. **14**, 1559-1567 DOI: 10.3390/microbiolres14040107.
262. Aldejohann, A.M., et al., *Rise in Candida Auris Cases and First Nosocomial Transmissions in Germany*. Dtsch Arztebl Int, 2023. **120**(27-28): p. 447-478.
263. Alshahrani, F.S., et al., *Description of Candida auris Occurrence in a Tertiary Health Institution in Riyadh, Saudi Arabia*. Healthcare (Basel), 2023. **11**(24).
264. Amadesi, S., et al., *Clonal Dissemination of Candida auris Clinical Isolates in Northern Italy, 2021*. Microb Drug Resist, 2023.

265. Amer, H.A., et al., *Characteristics and Mitigation Measures of Candida auris Infection: Descriptive Analysis from a Quaternary Care Hospital in Saudi Arabia, 2021-2022.* J Epidemiol Glob Health, 2023. **13**(4): p. 825-830.
266. Arenas, S.P., et al., *Persistent colonization of Candida auris among inpatients rescreened as part of a weekly surveillance program.* Infect Control Hosp Epidemiol, 2023: p. 1-4.
267. Ashkenazi-Hoffnung, L. and C. Rosenberg Danziger, *Navigating the New Reality: A Review of the Epidemiological, Clinical, and Microbiological Characteristics of Candida auris, with a Focus on Children.* J Fungi (Basel), 2023. **9**(2).
268. Ben Abid, F., et al., *Molecular characterization of Candida auris outbreak isolates in Qatar from patients with COVID-19 reveals the emergence of isolates resistant to three classes of antifungal drugs.* Clin Microbiol Infect, 2023. **29**(8): p. 1083.e1-1083.e7.
269. Benedict, K., et al., *Candida auris–Associated Hospitalizations, United States, 2017-2022.* Emerg Infect Dis, 2023. **29**(7): p. 1485-1487.
270. Biran, R., et al., *Nationwide Outbreak of Candida auris Infections Driven by COVID-19 Hospitalizations, Israel, 2021-2022.* Emerg Infect Dis, 2023. **29**(7): p. 1297-1301.
271. Byun, S.A., et al., *Virulence Traits and Azole Resistance in Korean Candida auris Isolates.* J Fungi (Basel), 2023. **9**(10).
272. Ceballos-Garzon, A., et al., *Emergence and circulation of azole-resistant C. albicans, C. auris and C. parapsilosis bloodstream isolates carrying Y132F, K143R or T220L Erg11p substitutions in Colombia.* Front Cell Infect Microbiol, 2023. **13**: p. 1136217.
273. Chew, K.L., et al., *Genomic epidemiology of human candidaemia isolates in a tertiary hospital.* Microb Genom, 2023. **9**(7).
274. Codda, G., et al., *In vivo evolution to echinocandin resistance and increasing clonal heterogeneity in Candida auris during a difficult-to-control hospital outbreak, Italy, 2019 to 2022.* Euro Surveill, 2023. **28**(14).
275. Cook, A., et al., *Neonatal invasive candidiasis in low- and middle-income countries: Data from the NeoOBS study.* Med Mycol, 2023. **61**(3).
276. de Melo, C.C., et al., *Colonized patients by Candida auris: Third and largest outbreak in Brazil and impact of biofilm formation.* Front Cell Infect Microbiol, 2023. **13**: p. 1033707.
277. de St Maurice, A., et al., *Clinical, microbiological, and genomic characteristics of clade-III Candida auris colonization and infection in southern California, 2019-2022.* Infect Control Hosp Epidemiol, 2023. **44**(7): p. 1093-1101.
278. Didik, T., et al., *Long-range air dispersion of Candida auris in a cardiothoracic unit outbreak in Hong Kong.* J Hosp Infect, 2023. **142**: p. 105-114.
279. El Zakhem, A., et al., *The Impact of COVID-19 on the Epidemiology and Outcomes of Candidemia: A Retrospective Study from a Tertiary Care Center in Lebanon.* J Fungi (Basel), 2023. **9**(7).
280. Erturk Sengel, B., et al., *Identification of Molecular and Genetic Resistance Mechanisms in a Candida auris Isolate in a Tertiary Care Center in Türkiye.* Mycopathologia, 2023. **188**(6): p. 929-936.
281. Escandón, P., et al., *Candida auris: a global pathogen that has taken root in Colombia.* Biomedica, 2023. **43**(Sp. 1): p. 278-287.

282. Fox-Lewis, S., et al., *Candida auris: lessons learnt from the first detected case in Aotearoa New Zealand.* N Z Med J, 2023. **136**(1580): p. 78-80.
283. Geremia, N., et al., *Candida auris as an Emergent Public Health Problem: A Current Update on European Outbreaks and Cases.* Healthcare (Basel), 2023. **11**(3).
284. Gorzalski, A., et al., *The use of whole-genome sequencing and development of bioinformatics to monitor overlapping outbreaks of Candida auris in southern Nevada.* Front Public Health, 2023. **11**: p. 1198189.
285. Goulart, M.A., et al., *Identification and infection control response to Candida auris at an academic level I trauma center.* Am J Infect Control, 2023.
286. Guchhait, P., B.N. Chaudhuri, and S. Das, *Bloodstream Infections with Opportunistic Pathogens in COVID-19 Era: A Real Challenge Necessitates Stringent Infection Control.* J Lab Physicians, 2023. **15**(1): p. 131-138.
287. Harris, A.D., et al., *Prevalence of Acinetobacter baumannii and Candida auris in Patients Receiving Mechanical Ventilation.* Jama, 2023. **330**(18): p. 1769-1772.
288. Harris, E., *CDC: Candida auris Fungal Infections and Drug Resistance on the Rise.* Jama, 2023. **329**(15): p. 1248.
289. Hassoun, N., et al., *Antifungal Use and Resistance in a Lower-Middle-Income Country: The Case of Lebanon.* Antibiotics (Basel), 2023. **12**(9).
290. Henriques, J., et al., *Candida auris in Intensive Care Setting: The First Case Reported in Portugal.* J Fungi (Basel), 2023. **9**(8).
291. Hong, H., et al., *Candida auris infection; diagnosis, and resistance mechanism using high-throughput sequencing technology: a case report and literature review.* Front Cell Infect Microbiol, 2023. **13**: p. 1211626.
292. Ito, Y., et al., *A case of fungal otitis externa caused by coinfection of Candida auris and Aspergillus flavus.* J Infect Chemother, 2023. **29**(8): p. 809-811.
293. Kaki, R., *Risk factors and mortality of the newly emerging Candida auris in a university hospital in Saudi Arabia.* Mycology, 2023. **14**(3): p. 256-263.
294. Katsiari, M., et al., *Emergence of Clonally-Related South Asian Clade I Clinical Isolates of Candida auris in a Greek COVID-19 Intensive Care Unit.* J Fungi (Basel), 2023. **9**(2).
295. Kekana, D., et al., *Candida auris Clinical Isolates Associated with Outbreak in Neonatal Unit of Tertiary Academic Hospital, South Africa.* Emerg Infect Dis, 2023. **29**(10): p. 2044-2053.
296. Koleri, J., et al., *Candida auris Blood stream infection- a descriptive study from Qatar.* BMC Infect Dis, 2023. **23**(1): p. 513.
297. Kozlova, O., et al., *Invasive Candidiasis in Adult Patients with COVID-19: Results of a Multicenter Study in St. Petersburg, Russia.* J Fungi (Basel), 2023. **9**(9).
298. Lee, E.H., et al., *Intrahospital transmission and infection control of Candida auris originating from a severely infected COVID-19 patient transferred abroad.* J Hosp Infect, 2023. **143**: p. 140-149.
299. Lyman, M., et al., *Worsening Spread of Candida auris in the United States, 2019 to 2021.* Ann Intern Med, 2023. **176**(4): p. 489-495.
300. Massic, L., et al., *Detection of five instances of dual-clade infections of Candida auris with opposite mating types in southern Nevada, USA.* Lancet Infect Dis, 2023. **23**(9): p. e328-e329.

301. McDougal, A.N., et al., *A cluster investigation of Candida auris among hospitalized incarcerated patients.* Antimicrob Steward Healthc Epidemiol, 2023. **3**(1): p. e244.
302. McGann, P., et al., *The Emergence and Persistence of Candida auris in Western New York With No Epidemiologic Links: A Failure of Stewardship?* Open Forum Infect Dis, 2023. **10**(3): p. ofad123.
303. Mulet Bayona, J.V., et al., *Candida auris from colonisation to candidemia: A four-year study.* Mycoses, 2023. **66**(10): p. 882-890.
304. Nahhal, S.B., et al., *Blood Stream Infections in COVID-19 Patients From a Tertiary Care Center in Lebanon: Causative Pathogens and Rates of Multi-Drug Resistant Organisms.* Mayo Clin Proc Innov Qual Outcomes, 2023. **7**(6): p. 556-568.
305. Noble, B.A., et al., *Candida auris rates in blood culture on the rise: results of US surveillance.* Microbiol Spectr, 2023. **11**(5): p. e0221623.
306. Ohashi, Y., et al., *The first case of clade I Candida auris candidemia in a patient with COVID-19 in Japan.* J Infect Chemother, 2023. **29**(7): p. 713-717.
307. Ortiz-Roa, C., et al., *Mortality Caused by Candida auris Bloodstream Infections in Comparison with Other Candida Species, a Multicentre Retrospective Cohort.* J Fungi (Basel), 2023. **9**(7).
308. Osaigbovo, II, et al., *The Nairobi Declaration 2023: A Commitment to Address Deadly Yet Neglected Fungal Diseases in Africa.* Med Mycol, 2023.
309. Oyardi, O., et al., *Phenotypic Investigation of Virulence Factors, Susceptibility to Ceragenins, and the Impact of Biofilm Formation on Drug Efficacy in Candida auris Isolates from Türkiye.* J Fungi (Basel), 2023. **9**(10).
310. Pandak, N., et al., *The Outcome of Antibiotic Overuse before and during the COVID-19 Pandemic in a Tertiary Care Hospital in Oman.* Antibiotics (Basel), 2023. **12**(12).
311. Prażyńska, M., et al., *Candida auris Infection in a Meningococcal Septicemia Survivor, Poland.* Mycopathologia, 2023. **188**(1-2): p. 135-141.
312. Rossi, A., et al., *Candida auris Discovery through Community Wastewater Surveillance during Healthcare Outbreak, Nevada, USA, 2022.* Emerg Infect Dis, 2023. **29**(2): p. 422-425.
313. Rowlands, J., et al., *Candida auris admission screening pilot in select units of New York City health care facilities, 2017-2019.* Am J Infect Control, 2023. **51**(8): p. 866-870.
314. Shuping, L., et al., *High Prevalence of Candida auris Colonization during Protracted Neonatal Unit Outbreak, South Africa.* Emerg Infect Dis, 2023. **29**(9): p. 1913-1916.
315. Simon, S.P., et al., *Comparative Outcomes of Candida auris Bloodstream Infections: A Multicenter Retrospective Case-Control Study.* Clin Infect Dis, 2023. **76**(3): p. e1436-e1443.
316. Singhal, T., et al., *Candida auris as the Predominant Species Causing Invasive Candidiasis in Neonates and Children.* Indian J Pediatr, 2023. **90**(9): p. 946.
317. Spettel, K., et al., *Candida auris in Austria-What Is New and What Is Different.* J Fungi (Basel), 2023. **9**(2).
318. Stanciu, A.M., et al., *First report of Candida auris in Romania: clinical and molecular aspects.* Antimicrob Resist Infect Control, 2023. **12**(1): p. 91.

319. Sticchi, C., et al., *Increasing Number of Cases Due to Candida auris in North Italy, July 2019-December 2022.* J Clin Med, 2023. **12**(5).
320. Walits, E. and S. Schaefer, *Outcome of Candida auris contact investigations conducted in a 6 month period at a New York City hospital.* Am J Infect Control, 2023.
321. Waters, A., et al., *Investigation of a Candida auris outbreak in a skilled nursing facility - Virginia, United States, October 2020-June 2021.* Am J Infect Control, 2023. **51**(4): p. 472-474.
322. Wong, S.C., et al., *Proactive infection control measures to prevent nosocomial transmission of Candida auris in Hong Kong.* J Hosp Infect, 2023. **134**: p. 166-168.
323. Xu, Z., et al., *A Candidemia Case Caused by a Novel Drug-Resistant Candida auris with the Y132F Mutation in Erg11 in Mainland China.* Infect Drug Resist, 2023. **16**: p. 3065-3072.
324. Munshi, A., et al., *Risk factors, antifungal susceptibility, complications, and outcome of Candida auris bloodstream infection in a tertiary care center in the western region of Saudi Arabia.* J Infect Public Health, 2024. **17**(1): p. 182-188.
